# Collaborative Reconstruction of PROPELLER‐EPI Data Using POCSMUSE (CORPUSE) for High‐Fidelity Diffusion MRI

**DOI:** 10.1002/mrm.70448

**Published:** 2026-05-30

**Authors:** Hailin Xiong, Liyuan Liang, Shihui Chen, Yi Li, Chenglang Yuan, Tianbaige Liu, Xiaorui Xu, Hing‐Chiu Chang

**Affiliations:** ^1^ Department of Biomedical Engineering The Chinese University of Hong Kong Hong Kong China; ^2^ Multi‐Scale Medical Robotics Center Hong Kong China; ^3^ Department of Diagnostic Radiology The University of Hong Kong Hong Kong China

**Keywords:** DTI, multishot DWI, Nyquist ghost correction, off‐resonance correction, PROPELLER‐EPI

## Abstract

**Purpose:**

To develop a reconstruction framework for DW‐PROPELLER‐EPI that improves image quality and SNR efficiency under per‐blade acceleration while minimizing EPI‐related artifacts, enabling high‐resolution diffusion‐tensor imaging (DTI) with fewer blades.

**Methods:**

We propose CORPUSE, a joint reconstruction framework adapted from POCSMUSE and tailored for multiblade DW‐PROPELLER‐EPI. The method integrates distortion‐correction operators into a joint‐blade reconstruction model and exploits the inherent redundancy of multiblade sampling. Leveraging self‐extracted composite 2D phase errors and blade‐specific field maps as physics‐based constraints, CORPUSE improves reconstruction performance. By using wider blades with higher per‐blade acceleration, the framework increases scan efficiency while preserving image quality and geometric fidelity. The framework was evaluated in healthy volunteers on a 1.5T MRI scanner across varying numbers of blades and with two PROPELLER‐EPI trajectories: long‐axis (LAP) and short‐axis (SAP), under different per‐blade accelerations and scan conditions.

**Results:**

In vivo experiments showed that CORPUSE improved image sharpness, geometric fidelity, and overall reconstruction quality compared with conventional PROPELLER‐EPI reconstruction. These improvements enabled higher per‐blade acceleration and wider blades without compromising image quality, thereby supporting more flexible and efficient reconstruction for LAP and SAP trajectory. Additionally, CORPUSE demonstrated greater motion resilience than multishot EPI based on multiplexed sensitivity encoding (MUSE), preserving structural detail even under subtle motion conditions.

**Conclusion:**

The CORPUSE framework enables high‐resolution, high‐quality DTI with fewer blades, improving the practicality of DW‐PROPELLER‐EPI. By maintaining SNR and geometric accuracy under high per‐blade acceleration, it offers a robust and efficient alternative to other multishot diffusion imaging approaches.

## 1 Introduction

Diffusion‐weighted imaging (DWI), including diffusion tensor imaging (DTI), is a powerful technique for probing tissue microstructure and molecular processes. It is widely used to detect pathological changes in intracranial diseases [1, 2, 3]. Ultrafast single‐shot echo‐planar imaging (ss‐EPI) is commonly used for DWI acquisition because of its high scan efficiency and strong immunity to motion. However, ss‐EPI has inherent drawbacks, including low spatial resolution and susceptibility‐induced distortions, which can compromise data quality.

Parallel imaging (PI) [4] has been used to improve ss‐EPI DWI, but geometric distortions still persist at modest acceleration (e.g., *R* = 2), and higher acceleration exacerbates noise amplification. While multishot EPI (ms‐EPI) can mitigate these limitations, motion‐induced intershot phase variations introduce aliasing artifacts. Although navigator echoes can estimate and correct these variations [5, 6], they reduce scan efficiency. Advanced self‐navigated ms‐EPI techniques, such as multiplexed sensitivity encoding (MUSE) [7] and projection onto convex sets reconstruction of multiplexed sensitivity encoded MRI (POCSMUSE) [8], have demonstrated effectiveness and robustness in achieving high‐quality brain DWI [9, 10, 11]. High signal‐to‐noise ratio (SNR) 3D encoding strategies (e.g., gSlider [12, 13]) enable high‐resolution DTI but are more sensitive to motion and require complex motion correction [14, 15]. In contrast, 2D acquisitions provide relatively superior motion robustness through slice‐wise sampling, offering a practical balance between SNR efficiency and artifact reduction. These advantages make 2D approaches particularly feasible for clinical applications.

Among the various 2D ms‐EPI techniques, periodically rotated overlapping parallel lines with enhanced reconstruction EPI (PROPELLER‐EPI) [16, 17] offers unique advantages through its radial‐like sampling scheme, which provides self‐navigated phase correction and inherent motion robustness. This technique has demonstrated high‐SNR and high‐resolution brain DWI and DTI, achieved by using a significant number of blades to oversample the *k*‐space center. However, this oversampling results in long scan time that limits clinical applicability. Reducing the number of blades improves efficiency but necessitates wider blades (i.e., longer echo train length; ETL), which exacerbates geometric distortions and image blurring due to off‐resonance and T2* decay effects [17]. While PI‐based per‐blade acceleration with additional distortion correction can mitigate these issues [18, 19], high acceleration factors introduce noise amplification and residual aliasing [20, 21], thereby limiting practical performance.

Iterative reconstruction of under‐sampled blades has been explored for conventional (non‐EPI) PROPELLER acquisitions [22, 23, 24, 25, 26] to alleviate PI‐related noise, but these approaches cannot be directly applied to PROPELLER‐EPI due to its unique challenges, including 2D Nyquist ghosts, off‐resonance effects within each blade, and diffusion‐induced inter‐blade phase variations. While POCS‐based methods can incorporate prior constraints to address motion and Nyquist ghosts, and the POCSMUSE framework effectively removes inter‐shot phase variations in multi‐shot diffusion imaging [8, 27, 28, 29, 30, 31], an approach that jointly handles all PROPELLER‐EPI–specific artifacts remains lacking. To address this gap, we propose CORPUSE, a POCSMUSE‐based collaborative reconstruction of PROPELLER‐EPI data. CORPUSE iteratively reconstructs images from fewer under‐sampled blades, while simultaneously correcting diffusion‐weighted EPI‐related artifacts and reducing noise amplification, thereby improving the SNR efficiency of PROPELLER‐EPI for diffusion MRI applications.

## 2 Theory

### 2.1 Signal Sampling in PROPELLER‐EPI

The *k*‐space data acquired in PROPELLER‐EPI with a phased‐array coil for the *n*th blade ($n=1\;\text{to}\;N_{b}$, where $N_{b}$ represents the total number of blades) at a rotation angle of (n−1)·θ are affected by B0 inhomogeneity and eddy‐current–induced Nyquist ghost errors. The B0 inhomogeneity introduces a phase accumulation proportional to the known field map ϕ(x,y) and the EPI echo spacing ΔT, resulting in a linearly increasing phase term along consecutive $k_{y}$ lines. Eddy currents further cause inconsistencies between odd and even echoes, producing blade‐dependent 2D phase errors. Consequently, the measured *k*‐space signals can be separated into odd and even components:

(1) $$\left\{\begin{array}{l} S_{m,n}^{o}\left(t_{x},t_{y}\right)\iint C_{m}\left(x,y\right)\cdot I\left(x,y\right)\cdot e^{-i\partial_{n}^{o}\left(x,y\right)}\cdot e^{-i2\pi \phi \left(x,y\right)\cdot \left(n_{y}\cdot ∆T\right)}\\ \;\cdot e^{-i2\pi \left[k_{x}\left(t_{x}\right)x+k_{y}\left(t_{y}\right)y\right]}\text{dxdy}\\ S_{m,n}^{e}\left(t_{x},t_{y}\right)\iint C_{m}\left(x,y\right)\cdot I\left(x,y\right)\cdot e^{-i\partial_{n}^{e}\left(x,y\right)}\cdot e^{-i2\pi \phi \left(x,y\right)\cdot \left(n_{y}\cdot ∆T\right)}\\ \;\cdot e^{-i2\pi \left[k_{x}\left(t_{x}\right)x+k_{y}\left(t_{y}\right)y\right]}\text{dxdy} \end{array}\right.$$

Here, $S_{m,n}$ denotes the data for the *n*th blade measured from the *m*th coil element ($m=1\;\text{to}\;N_{c}$, where $N_{c}$ denotes the total number of coil elements), $C_{m}$is the coil sensitivity profile for the *m*th coil element, and I is the reconstructed complex image. Additionally, $\partial_{n}^{o}\left(x,y\right)$ and $\partial_{n}^{e}\left(x,y\right)$ denote the odd‐ and even‐echo phase errors specific to each blade. The rotated EPI trajectory for the *n*th blade is given by:

(2) $$k_{x}\left(t_{x}\right)=∆k_{x}\cdot n_{x}\cos\left(\left(n-1\right)\cdot \theta \right)-∆k_{y}\cdot n_{y}\sin\left(\left(n-1\right)\cdot \theta \right)$$

(3) $$\begin{array}{c} k_{y}\left(t_{y}\right)=∆k_{x}\cdot n_{x}\sin\left(\left(n-1\right)\cdot \theta \right)+∆k_{y}\cdot n_{y}\cos\left(n-1\right)\cdot \theta ) \end{array}$$

where $k_{x}\left(t_{x}\right)$ and $k_{y}\left(t_{y}\right)$represent the instantaneous *k*‐space coordinates. The EPI sampling indices $\left(n_{x},n_{y}\right)$ along the frequency‐encoding (FE) and phase‐encoding (PE) directions fall within the ranges $-\frac{N_{\mathrm{FE}}}{2}\leq n_{x}\leq \frac{N_{\mathrm{FE}}}{2}$ and $-\frac{N_{\mathrm{PE}}}{2}\leq n_{x}\leq \frac{N_{\mathrm{PE}}}{2}$, respectively, where $N_{\mathrm{FE}}$ and $N_{\mathrm{PE}}$ denote the number of sampling points along the FE and PE directions.

The association of diffusion‐encoding gradients and macroscopic brain motion introduces an additional interblade phase variation $\delta_{n}\left(x,y\right)$. This combines with the odd/even phase errors related to the Nyquist ghost artifact to yield the following composite phase error terms:

(4) $$\begin{array}{c} \left\{\begin{array}{c} \partial_{n}^{\prime o}\left(x,y\right)=\partial_{n}^{o}\left(x,y\right)+\delta_{n}\left(x,y\right)\\ {\partial^{\prime }}_{n}^{e}\left(x,y\right)=\partial_{n}^{e}\left(x,y\right)+\delta_{n}\left(x,y\right) \end{array}\;\right. \end{array}$$

Hence, the odd and even signal samplings of all acquired blades can be combined to form a linear system, incorporating data inconsistencies represented in the following matrix form:

(5) $$\begin{array}{c} \left[\begin{array}{c} \begin{array}{c} S_{1}^{o}\\ \vdots \\ S_{n}^{o} \end{array}\\ \begin{array}{c} S_{1}^{e}\\ \vdots \\ S_{n}^{e} \end{array} \end{array}\right]=\left[\begin{array}{c} \begin{array}{c} E_{1}^{o}\left(C,\phi_{1},\partial_{1}^{\prime o}\right)\\ \vdots \\ E_{n}^{o}\left(C,\phi_{n},\partial_{n}^{\prime o}\right) \end{array}\\ \begin{array}{c} E_{1}^{e}\left(C,\phi_{1},\partial_{1}^{\prime e}\right)\\ \vdots \\ E_{n}^{e}\left(C,\phi_{n},\partial_{n}^{\prime e}\right) \end{array} \end{array}\right]I⟹S=E\left(C,\phi ,\partial^{\prime }\right)I \end{array}$$

where $E\left(C,\phi ,\partial^{\prime }\right)$ represents the encoding matrix incorporating the Fourier transform (with the sampling operator defined by the rotating EPI trajectory), coil sensitivity profile (C), phase modulation (ϕ) due to off‐resonance, and composite 2D phase errors $\left(\partial^{\prime }\right)$. Theoretically, the image (I) can be reconstructed by performing matrix inversion of the Equation (5). However, the encoding matrix has a size on the order of $N_{\mathrm{FE}}\times N_{\mathrm{PE}}\times N_{C}\times N_{b}$, making direct inversion numerically ill‐conditioned and computationally prohibitive [32]. Therefore, practical implementations rely on approximate methods or iterative solvers to address the challenge of collaborative multi‐blade reconstruction in PROPELLER‐EPI.

### 2.2 Collaborative Framework for CORPUSE

**FIGURE 1:**
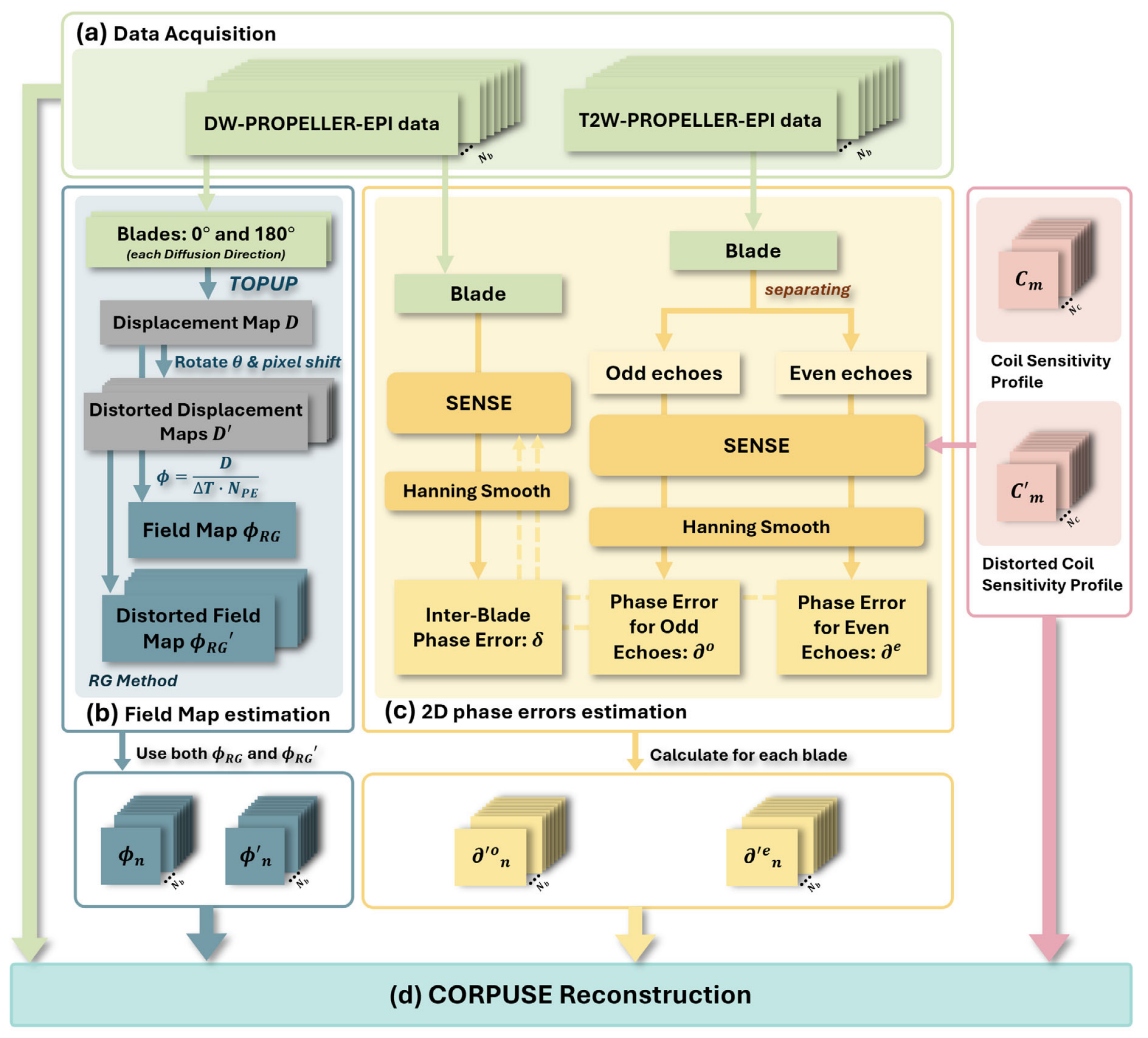
Flowchart of the CORPUSE reconstruction framework comprising three main steps. Step 1 involves (a) PROPELLER‐EPI data acquisition. Step 2 performs data pre‐processing to calculate reconstruction constraints, including (b) field map estimation using self‐referenced PROPELLER‐EPI blade pairs, and (c) composite 2D phase error estimation incorporating Nyquist ghost phase errors and inter‐blade phase variations. Step 3 executes the (d) collaborative CORPUSE reconstruction, combining PROPELLER‐EPI data from Step 1 with reconstruction constraints from Step 2. This flowchart shows a fully self‐referenced CORPUSE workflow, where all constraints are derived exclusively from PROPELLER‐EPI data without requiring additional calibration scans.

Building upon the principles of the previously developed POCSMUSE method for handling data inconsistencies in multishot DWI, we propose CORPUSE, a collaborative reconstruction framework tailored for multi‐blade data. CORPUSE jointly reconstructs all blades by exploiting their inherent geometric consistency, in contrast to conventional approaches [17] that reconstruct under‐sampled blades individually before final combination. Similar to POCSMUSE, CORPUSE retains the core strategy of joint reconstruction with phase estimation to resolve inter‐shot inconsistencies within a POCS‐based iterative framework. It extends this framework to accommodate DW‐PROPELLER‐EPI data by incorporating rotation operators for multi‐blade reconstruction and explicitly modeling blade‐dependent off‐resonance effect together with composite 2D phase errors, which are not accounted for in standard MUSE or POCS‐MUSE. The required parameters in Equation (5) are extracted through several pre‐processing steps using only the multiblade data, eliminating the need for additional reference scans or navigators. Figure 1 illustrates the overall flowchart of CORPUSE, including three main steps: first, acquisition parameter design; second, preprocessing to estimate field maps and composite 2D phase errors; and third, collaborative reconstruction of all blades using CORPUSE framework. Details of each step are described in the following method section.

## 3 Methods

All MRI experiments were performed on a 1.5T MRI scanner (Artist, GE Healthcare). This study was approved by the local Institutional Review Board (IRB), and three human subjects were included. Detailed acquisition and reconstruction procedures are described below.

### 3.1 Data Acquisition

A 2D diffusion‐weighted PROPELLER‐EPI (DW‐PROPELLER‐EPI) pulse sequence is used to acquire T_2_‐weighted (at *b*‐value = 0 s/mm^2^), and diffusion‐weighted blades. The total scan time for PROPELLER‐EPI is mainly dominated by $N_{b}$ (equivalent to the number of shots in multishot DWI acquisitions). For a 360° circular *k*‐space coverage with a given $N_{b}$, the rotation angle (θ) is equal to $\left(360{}^{\circ}/N_{b}\right)$. The blade sizes are defined by N×L, where $N=N_{\mathrm{FE}}$ and $L=N_{\mathrm{PE}}$. The blade width (L) (i.e., number of PE lines for LAP‐EPI acquisition) can be determined using the following formula:

(6) $$\begin{array}{c} L=f\left(\frac{N\times \pi }{N_{b}}\right) \end{array}$$

Here, y≔f(x) denotes the greatest even integer ≤ x.

Accordingly, to reduce the scan time, fewer blades (i.e., reduced $N_{b}$) can be used for data acquisition while increasing the blade width (L) to ensure gapless *k*‐space coverage. However, this approach reduces both the angular sampling density [33] (i.e., increased angular separation between blades) and the *k*‐space oversampling factor, potentially degrading spatial resolution and diminishing SNR. This degradation manifests as stronger radial streaking in the point‐spread function (PSF) (Figure S1). Wider blade (i.e., increased PE lines) also extends the EPI ETL, exacerbating off‐resonance effects and image blurring to address these challenges associated with reduced $N_{b}$, undersampling of PE lines within each blade can be employed, and the collaborative multi‐blade reconstruction of the CORPUSE framework can further suppress off‐resonance artifacts and noise amplification.

### 3.2 Field Map Estimation

In PROPELLER‐EPI, orientation‐dependent distortions along the PE direction of each blade can cause blurring in reconstructed images. Conventional reconstruction [17] applies multichannel phase modulation (MPM) [34] to correct blade‐specific pixel shifts using distorted field maps, followed by triangular $k_{y}$‐weighting to suppress residual off‐resonance effects before multiblade combination. In CORPUSE, these off‐resonance correction operators are incorporated directly into the iterative reconstruction, enabling simultaneous off‐resonance correction across all blades. The field maps used for off‐resonance correction are estimated independently for each diffusion direction to account for direction‐dependent variations due to eddy current effects.

The 360° *k*‐space coverage permits self‐referenced estimation of the intrinsic undistorted B0 field map from displacement maps calculated using the TOPUP‐based [35, 36] reversed gradient (RG) method [37, 38], leveraging blade pairs with opposite PE directions [39]. For each slice and diffusion direction, reconstructed blade images with opposite PE directions (e.g., 0° and 180°) were selected as a standard reversed‐gradient pair and provided to FSL TOPUP to estimate a displacement map, which was used for all subsequent calculations of blade‐specific field maps and distortion correction within CORPUSE. Although each blade pair can independently be used to generate an undistorted field map, this single reversed‐gradient pair (e.g., 0° and 180°) produces an undistorted field map with accuracy comparable to those derived from multiple opposing pairs (a detailed comparison is provided in Figure S2). For this work, the displacement map (D) estimated from this single blade pair is therefore used to generate a distorted displacement map ($D^{\prime }$) by applying the pixel shifts defined by D to simulate the geometric distortion specific to the distinct rotation angle of each blade. The undistorted and distorted field maps ($\phi_{\mathrm{RG}}$ and $\phi_{\mathrm{RG}}^{\prime }$) are subsequently derived from D and $D^{\prime }$, respectively, according to the following equation:

(7) $$\begin{array}{c} \phi_{\mathrm{RG}}\left(x,y\right)=\frac{D\left(x,y\right)}{∆T\cdot N_{\mathrm{PE}}} \end{array}$$

where ∆T is the effective echo‐spacing and $N_{\mathrm{PE}}$ is the number of sampled PE lines.

If the multi‐blade configuration lacks opposite PE directions (e.g., only 180° *k*‐space coverage), conventional field mapping based on multiecho gradient‐echo imaging (ME‐GRE) [34, 40] can provide an alternative method for measuring an undistorted field map ($\phi_{\mathrm{ME}}$). Specifically, ME‐GRE images first underwent complex‐value coil combination. The coil‐combined ME‐GRE images were then processed using voxel‐wise temporal phase unwrapping and linear fitting of phase evolution across echoes, with spatial smoothing applied using a 2D Hanning window to improve robustness. The field map was estimated as:

(8) $$\begin{array}{c} \phi_{\mathrm{ME}}\left(x,y\right)=\left(\frac{∆\arg_{\mathrm{TE}}\left(x,y\right)}{∆\mathrm{TE}}\right) \end{array}$$

where $∆\arg_{\mathrm{TE}}\left(x,y\right)$ represents the slope of the linearly fitted phase evolution across echoes, and ∆TE is the echo time interval. The distorted field maps ($\phi_{{\mathrm{ME}}_{n}}^{\prime }$) for all blades were then generated from $\phi_{\mathrm{ME}}$ using the same process as described previously for generating $\phi_{{\mathrm{RG}}_{n}}^{\prime }$.

### 3.3 Estimation of Composite 2D Phase Errors

In conventional PROPELLER‐EPI reconstruction [17], Nyquist ghost and inter‐blade phase variations (induced by diffusion encoding) are corrected sequentially for each blade to prevent destructive interference after multi‐blade combination (e.g., signal shading in reconstructed images). Nyquist‐ghost correction typically requires blade‐specific 2D phase maps derived from reference scans. To improve scan efficiency, we adopt a self‐referenced 2D Nyquist‐ghost estimation strategy (Figure 1c). Specifically, for each T2‐weighted blade with acceleration factor R, *k*‐space is separated into odd and even echoes (i.e., $S_{m,n}^{o}$ and $S_{m,n}^{e}$), yielding an effective acceleration of 2R. Subsequently, each subset is reconstructed using SENSE with a distorted coil sensitivity profile ($C_{m,n}^{\prime }$), which is obtained by warping the reference GRE‐derived sensitivity maps using the blade‐specific field maps to match the distorted geometry of each individual blade. The blade‐specific 2D Nyquist ghost phase errors (i.e., $\partial_{n}^{o}$ and $\partial_{n}^{e}$) are then estimated directly from the SENSE‐reconstructed odd and even images. For diffusion‐weighted blades, SENSE reconstruction is performed using the corresponding $C_{m,n}^{\prime }$ and 2D Nyquist ghost phase errors derived from the T2‐weighted data [41]. Diffusion‐induced inter‐blade phase variations (i.e., $\delta_{n}$) are then measured separately from the SENSE‐reconstructed diffusion‐weighted images. Spatial 2D Hanning window smoothing is applied to these direction‐specific phase maps using a 20 × 20 kernel to suppress high frequency noise, yielding blade‐specific composite 2D phase errors ($\partial_{n}^{\prime o}$ and $\partial_{n}^{\prime e}$), which combine both Nyquist ghost and diffusion‐related components.

### 3.4 CORPUSE Reconstruction

**FIGURE 2:**
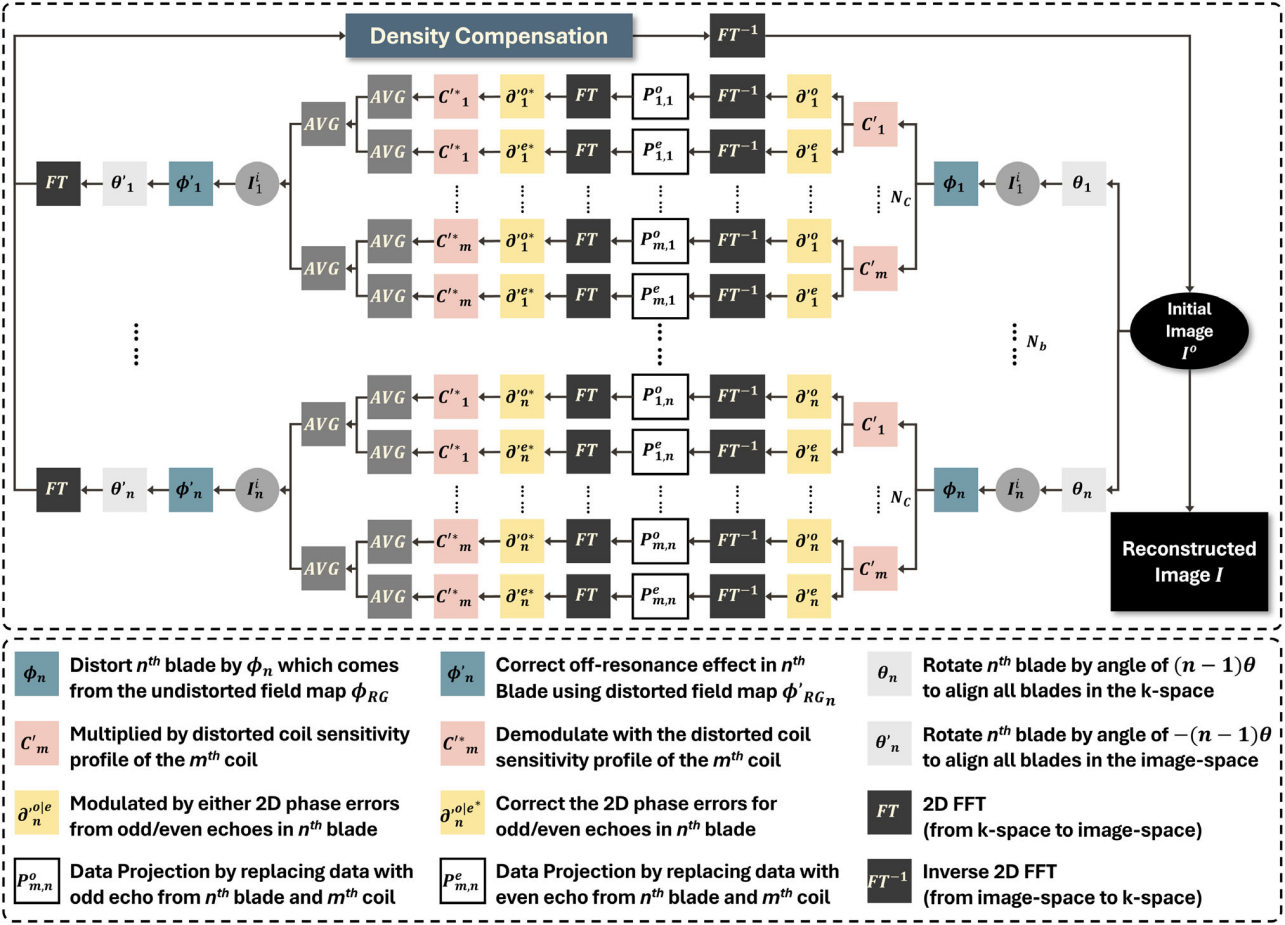
The iterative CORPUSE reconstruction framework, adapted from the original POCSMUSE framework, incorporates rotation and off‐resonance correction operators while integrating blade‐specific distorted coil sensitivity profiles, field maps and composite 2D phase errors as reconstruction constraints. A detailed description of each operator is provided in the panel below. This framework can accommodate both long‐axis and short‐axis PROPELLER‐EPI (LAP‐EPI and SAP‐EPI) through distinct data projection strategies. For Partial‐Fourier acquisitions, similar to the approach with adaptive Partial‐Fourier reconstruction [42], k‐space data are initially multiplied by a ramp‐weighting prior to iteration.

Following the extraction of all necessary information through the pre‐processing steps described above, the final PROPELLER‐EPI image is collaboratively reconstructed from all blades using the CORPUSE reconstruction framework. Figure 2 illustrates the CORPUSE reconstruction framework, adapted from the original POCSMUSE by incorporating several key constraints, including a rotation operator, an off‐resonance correction operator, blade‐specific distorted coil sensitivity profiles, and composite 2D phase errors. Prior to reconstruction, the *k*‐space data of all blades are zero‐padded (doubling the sampled data points) to enhance the reconstructed resolution. Then, the CORPUSE reconstruction is performed iteratively through the following steps.

*Step 1*: A zero matrix serves as the initial input image $I^{i}\;\left(\text{where}\;i=0\;\mathrm{at}\;1\mathrm{st}\;\text{iteration}\right)$, which is replicated to generate ${I^{i}}_{n}$ using rotation operators with the rotation angles corresponding to each blade.

*Step 2*: Each ${I^{i}}_{n}$ undergoes distortion using an off‐resonance correction operators with blade‐aligned undistorted field maps $\phi_{{\mathrm{RG}}_{n}}$, which are rotated versions of the original undistorted field map $\phi_{\mathrm{RG}}$, to simulate distortions along PE direction.

*Step 3*: The distorted blade images are multiplied by their corresponding distorted coil sensitivity profiles ($C_{m,n}^{\prime }$).

*Step 4*: The multicoil distorted blade images are further modulated by either $\partial_{n}^{\prime o}$ or $\partial_{n}^{\prime e}$ to generate two datasets incorporating composite 2D phase errors for odd and even echoes.

*Step 5*: All multicoil distorted blade images are transformed into *k*‐space where data projections ($P_{m,n}^{o}$and $P_{m,n}^{e}$) are performed for odd and even echoes using the acquired blade data (i.e., $S_{m,n}^{o}$ and $S_{m,n}^{e}$).

*Step 6*: Projected *k*‐space data are inverse‐transformed to image space (i.e., ${I^{o}}_{m,n}^{i}$ and ${I^{e}}_{m,n}^{i}$) with correction of the composite 2D phase errors for odd and even echoes. These are then averaged to generate coil‐combined blade images ($I_{n}^{i+1}$) with the combination of odd and even echoes using the following equation:

(9) $$\begin{array}{c} I_{n}^{i+1}=\sum_{m=1}^{N_{c}}\frac{C_{m}^{\prime *}{\partial_{n}^{\prime o}}^{*}}{\sum_{m=1}^{N_{c}}{\left|C_{m}^{\prime }\right|}^{2}\left|\partial_{n}^{\prime o}\right|}\;{I^{o}}_{m,n}^{i}+\sum_{m=1}^{N_{c}}\frac{C_{m}^{\prime *}\partial_{n}^{\prime e*}}{\sum_{m=1}^{N_{c}}{\left|C_{m}^{\prime }\right|}^{2}\left|\partial_{n}^{\prime e}\right|}\;{I^{e}}_{m,n}^{i} \end{array}$$

where $C_{m}^{\prime *}$and $\partial_{n}^{\prime *}$ represent the complex conjugates of $C_{m}^{\prime }$ and $\partial_{n}^{\prime }$, respectively.

*Step 7*: Each coil‐combined blade image ($I_{n}^{i+1}$) undergoes an off‐resonance correction operatorsusing the distorted field map $\phi_{\mathrm{RG}}^{\prime }$ to correct the off‐resonance effects.

*Step 8*: All blade images are spatially aligned using rotation operators according to the corresponding rotation angles.

*Step 9*: All aligned blade images are transformed into *k*‐space and combined using density compensation, as employed in conventional PROPELLER‐EPI reconstruction.

*Step 10*: The combined *k*‐space is inverse‐transformed to image space to produce a reconstructed PROPELLER‐EPI image ($I^{i+1}$) for the next iteration.

*Step 11*: The procedures described above are iterated until the reconstructed PROPELLER‐EPI image converges (typically until $\left\|I^{i+1}-I^{i}\right\|<\varepsilon$).

### 3.5 Data Simulation and Phantom Study

To compare conventional PROPELLER‐EPI and CORPUSE reconstruction, we first evaluated PSF characteristics and robustness to varying B0 inhomogeneity using simulations. A 12‐channel 2D receiver coil was modeled, and a 128 × 128 circular phantom was multiplied by the coil sensitivities to form multicoil data. This dataset was replicated and rotated to generate 24 PROPELLER blades (blade size = 128 × 32; rotation increment = 15°). Before Fourier transformation, only the central 32 PE lines were retained to simulate LAP‐EPI acquisition. T2* decay (T2* = 40 ms) and an echo spacing of 0.8 ms were applied, and linear field maps with maximum inhomogeneity ranging from 50 to 300 Hz were used to introduce off‐resonance effects.

To complement the simulations, two water‐phantom datasets (Scan 3, Table 1) were acquired under different shimming conditions (auto vs. deliberately poor shimming) to induce varying B0 inhomogeneity. Auto shimming was applied to partially compensate for field inhomogeneity associated with susceptibility effect between tissues, while poor shimming was applied to create severe field inhomogeneity. Field maps for both settings were obtained using ME‐GRE (TE1/TE2/TE3 = 2.7/6.8/11 ms; TR = 100 ms). All simulated and acquired datasets were reconstructed using both conventional PROPELLER‐EPI reconstruction and the CORPUSE framework.

**TABLE 1:** Primary scan parameters for long‐axis DW‐PROPELLER‐EPI acquisitions.

Scan	1	2	3	4	5	6	7	8	9	10	11	12
# of blades ($N_{b}$)	6	18	24	18	12	6	8	8	8	8	8	24
Rotation angle (θ)	60°	20°	15°	20°	30°	60°	45°	45°	45°	45°	45°	15°
FOV (cm)	24	24	22	22	22	22	22	22	22	22	24	22
Blade size (N×L)	192 × 100	192 × 32	192 × 32	192 × 32	192 × 50	192 × 100	192 × 72	192 × 72	192 × 72	192 × 72	192 × 72	280 × 36
PF factor	0.74	1	1	1	1	0.74	1	1	1	1	1	1
ETL	74	32	32	32	50	74	72	72	72	72	72	36
Acceleration factor (R)	1	1	1	1	1	1	1	2	3	4	2	1
TE (ms)	89.4	77.7	82.6	82.6	96.4	94.8	113	84.5	76.5	73.7	65.3	101
*b* (s/mm^2^)	0, 800	0, 800	0, 1000	0, 1000	0, 1000	0, 1000	0, 1000	0, 1000	0, 1000	0, 1000	0, 800	0, 1500
# diffusion direction for *b*‐value > 0 s/mm^2^	3	3	3 + 1^a^	3 + 1^a^	3 + 1^a^	3 + 1^a^	3 + 1^a^	3 + 1^a^	3 + 1^a^	3 + 1^a^	15	3
Scan time^b^ (s)	18	54	72	54	36	18	24	24	24	24	24	72

^a^ 3 + 1 diffusion direction means first scan with three diffusion directions and a repeated scan for one diffusion direction.

^b^ Scan Time for each diffusion direction.

### 3.6 In Vivo Data Acquisitions

**FIGURE 3:**
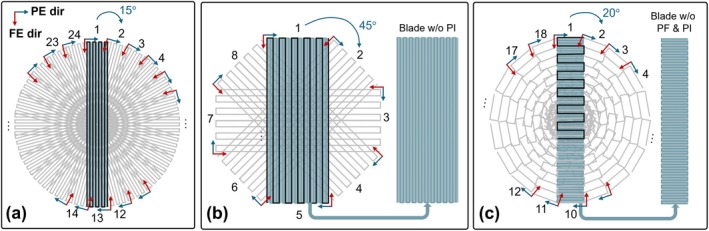
Exemplary trajectories of PROPELLER‐EPI for (a) 24‐blade LAP‐EPI, (b) 8‐blade LAP‐EPI with a per‐blade acceleration factor of 2, and (c) 18‐blade SAP‐EPI with Partial‐Fourier acquisition and a per‐blade acceleration factor of 3. ETLs increase when fewer blades are acquired in LAP‐EPI or in SAP‐EPI. Longer ETL often results in prolonged echo time (TE) and degraded image quality due to T2* decay and off‐resonance effects. To shorten TE, PI and Partial Fourier techniques can be applied to PROPELLER‐EPI, as illustrated in (b) and (c), respectively. Additionally, 360° k‐space coverage allows for the pairing of blades with opposite PE directions, as demonstrated by Blades 1 and 13 in (a), Blades 1 and 5 in (b), and Blades 1 and 10 in (c).

A total of 14 in vivo brain DW‐PROPELLER‐EPI datasets, comprising both long‐axis (LAP‐EPI) and short‐axis (SAP‐EPI) [43] acquisitions, were acquired from three healthy subjects to evaluate the reconstruction performance and artifact reduction capabilities of the proposed CORPUSE framework. All acquisitions were performed with a repetition time (TR) of 3000 ms and a slice thickness of 5 mm. Other primary scan parameters used to achieve a target reconstruction matrix of 192 × 192 for all Scans except 280 × 280 for Scan 12 in Table 1, with 360° *k*‐space coverage, including a varying number of blades and their corresponding blade sizes, are detailed in Tables 1 and S1. Representative trajectories for Scans 3, 8 in Table 1, and Scan 6 in Table S1 are illustrated in Figure 3. Additionally, an ME‐GRE sequence was used to measure a field map. This field map was used to compare the off‐resonance correction performance of the CORPUSE framework against a field map derived from the blade pair using RG method. The ME‐GRE data were acquired at the same reconstruction resolution as the DW‐PROPELLER‐EPI data, using three echoes (TE1/TE2/TE3 = 2.7/6.8/11 ms) and a TR of 100 ms. All data reconstructions and analyses were performed in MATLAB (The MathWorks, Natick, MA). Detailed descriptions of the evaluations for each scan are provided in subsequent sections.

### 3.7 Assessment of Prior Constraints

In addition to interblade phase variations (analogs to the intershot phase variations incorporated in POCUMUSE), CORPUSE includes two additional constraints: off‐resonance effects and 2D Nyquist ghost phase errors. The influence of these constraints on reconstruction performance was systematically evaluated.

First, the effectiveness of off‐resonance correction operators in correcting for off‐resonance effects was investigated using two different types of field maps: $\phi_{\mathrm{ME}}$, estimated from ME‐GRE data, and $\phi_{\mathrm{RG}}$, estimated using FSL TOPUP [35, 36] from blade pairs acquired with identical diffusion encoding directions. Specifically, $\phi_{\mathrm{RG}}$ provide a direction‐specific field map for each diffusion direction. DW‐PROPELLER‐EPI data were reconstructed using both the conventional method and CORPUSE, each combined with either $\phi_{\mathrm{ME}}$or $\phi_{\mathrm{RG}}$. A baseline reconstruction without off‐resonance correction was also included. Acquisition parameters for these LAP‐EPI datasets correspond to Scans 1 and 2 in Table 1.

Second, to evaluate the effectiveness of ghost suppression, DW‐PROPELLER‐EPI data were reconstructed using CORPUSE with and without the 2D Nyquist ghost constraint. Specifically, the condition without ghost correction was achieved by solely incorporating the inter‐blade phase variation $\delta_{n}$ in the iteration, separating odd and even echoes. A baseline reconstruction using the conventional PROPELLER‐EPI method without ghost correction was also generated for direct comparison. Off‐resonance correction was disabled for all reconstructions in this assessment to isolate the impact of ghost suppression on final image quality. The acquisition parameters for these datasets correspond to Scan 1 in Table 1. Ghost suppression performance was quantified using the ghost‐to‐signal ratio (GSR), defined as the ratio of the mean signal in four ghost regions of interest (ROIs) to that in a central brain ROI.

### 3.8 Assessment of CORPUSE Reconstruction With Different Scan Parameters

#### 3.8.1 Data Acquisition

A 24‐blade DW‐PROPELLER‐EPI dataset (Scan 3, Table 1) with a blade size of 192 × 32 was acquired using LAP‐EPI to serve as a high–angular‐density reference. To evaluate performance at shorter scan time, three additional DW‐PROPELLER‐EPI datasets were acquired using LAP‐EPI with 18, 12, and 6 blades (Scans 4–6 in Table 1). For the acquisitions with less than or equal to 12 blades, the blade width was enlarged to retain gapless *k*‐space coverage. As a result, Partial‐Fourier and PI were applied to minimize TE prolongation. Specifically, a Partial‐Fourier factor of 0.74 was used for the six‐blade acquisition (Scan 6 in Table 1). Additionally, per‐blade acceleration factors *R* = 1–4 were applied to four separate eight‐blade acquisitions (Scans 7–10 in Table 1). For all acquisitions described above, an additional repeated scan of one diffusion direction was acquired to facilitate SNR estimation. To demonstrate the feasibility of the CORPUSE framework for DTI, an additional eight‐blade LAP‐EPI dataset with 15 diffusion directions was acquired (Scan 11 in Table 1).

Furthermore, to evaluate the applicability of the proposed CORPUSE framework to different DW‐PROPELLER‐EPI trajectories, several datasets were acquired using SAP‐EPI [43] (Scans 1–7 in Table S1). Additional experiments were performed to investigate the influence of acquisition parameters on SAP‐EPI reconstruction, including variations in per‐blade acceleration (*R* = 1–3; Scans 1–3 in Table S1), blade width (32 × 192, 50 × 192, and 72 × 192; Scans 3–5 in Table S1), and number of blades (18, 12, and 6 blades) under accelerated acquisition with *R* = 3 (Scans 3, 6, 7 in Table S1). Owing to the inherently longer ETL for each blade in SAP‐EPI, a Partial‐Fourier factor of 0.63 was used to reduce TE and mitigate off‐resonance effects.

To evaluate performance at high‐b‐value and high‐resolution, as well as motion robustness, two 24‐blade LAP‐EPI datasets (Scan 12 in Table 1) were acquired: one without motion and one with subtle head motion (±5° rotation every 15 s during the second half of the scan). For an acquisition time–matched comparison, two additional four‐shot MUSE DWI datasets were collected at the same b‐value and under the same motion conditions, using six signal averages (number of excitations, NEX = 6). The scan parameters for four‐shot MUSE DWI acquisition were: matrix size = 280 × 280, TR/TE = 3000/98.2 ms, and Partial‐Fourier factor = 0.72.

#### 3.8.2 Data Reconstruction and Analysis

All PROPELLER‐EPI datasets were reconstructed using either conventional PROPELLER‐EPI reconstruction [17, 43] or the CORPUSE framework. Direction‐specific field maps derived from blade pairs were used for off‐resonance correction in both methods. For Partial‐Fourier reconstruction, ramp‐weighting was applied per blade prior to Homodyne reconstruction (in conventional PROPELLER‐EPI) or prior to iteration (in the CORPUSE framework) [42]. The final reconstructed images for the three orthogonal diffusion directions were then averaged to generate a mean DW image. Quantitative evaluation focused on the LAP‐EPI acquisitions with varying scan parameters (6–24 blades; *R* = 1–4 for eight‐blade acquisitions). SNR and SNR efficiency were used as performance metrics. SNR was defined as the ratio of the mean signal (averaged over 10 white‐matter ROIs) to the standard deviation of signal intensity across the two repeated scans. SNR efficiency was computed as:

(10) $$\begin{array}{c} {\mathrm{SNR}}_{\text{efficiency}}=\frac{{\mathrm{SNR}}_{n-\text{blade}}\times \sqrt{T_{24-\text{blade}}}}{{\mathrm{SNR}}_{24-\text{blade}}\times \sqrt{T_{n-\text{blade}}}}\% \end{array}$$

where $T_{n-\text{blade}}$ and $T_{24-\text{blade}}$denote the scan time of the *n*‐blade and 24‐blade reference DW‐PROPELLER‐EPI acquisitions. Statistical SNR comparisons were performed using two‐sample Student's *t*‐tests, with p<0.001 considered statistically significant.

For the eight‐blade LAP‐EPI dataset with 15 diffusion directions, both reconstruction pipelines produced full DTI datasets. Both datasets underwent identical post‐processing. First, all diffusion‐weighted images (including the *b* = 0 image) were denoised using a 2D adaptive Wiener filter with a 3 × 3 neighborhood. The diffusion tensor was then estimated voxel‐wise using in‐house MATLAB scripts with linear least‐squares fitting of the standard six‐parameter tensor model, from which traceDWI and color‐encoded FA (cFA) maps were generated for visual comparison. In addition, high‐resolution, high‐*b*‐value LAP‐EPI data also underwent both conventional reconstruction and CORPUSE, and their resulting images were compared with time‐matched four‐shot MUSE DWI data [7].

## 4 Results

### 4.1 Simulation and Phantom Results

**FIGURE 4:**
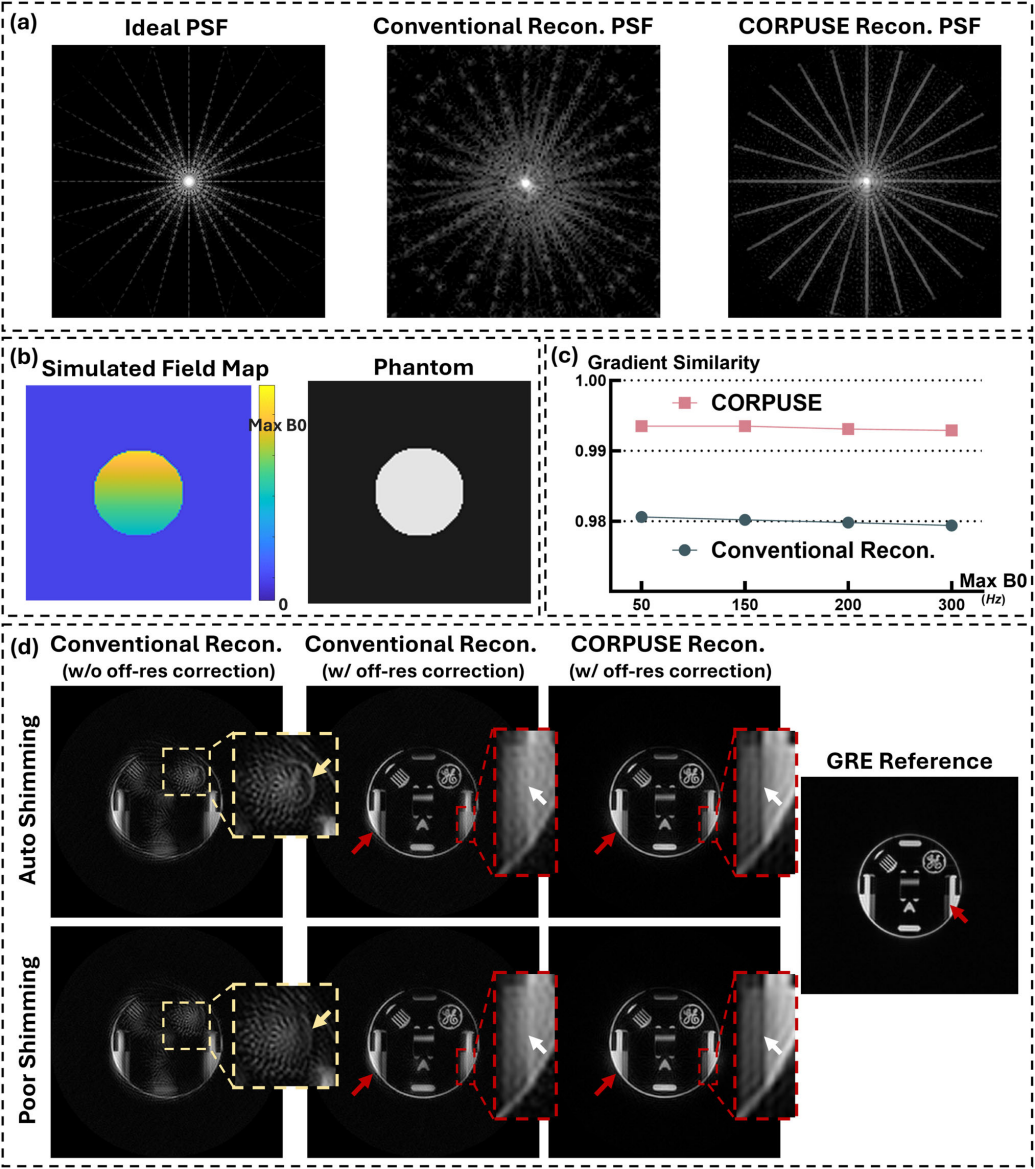
(a) Ideal point spread function (PSF) from the PROPELLER sampling trajectory, alongside reconstructed PSFs from conventional PROPELLER‐EPI reconstruction and CORPUSE using single‐point data, showing a markedly sharper PSF with CORPUSE. (b) Simulated linear B0 field map and circular phantom used to assess off‐resonance sensitivity. (c) Gradient similarity between reconstructed image and phantom image across all B0 levels. (d) Phantom experiments under auto and poor shimming, illustrating that CORPUSE preserves structural sharpness and fine detail more effectively than conventional PROPELLER‐EPI reconstruction; the image from first ME‐GRE echo is included as a structural reference.

Figure 4a compares the PSFs from conventional PROPELLER‐EPI reconstruction and the CORPUSE framework, showing that CORPUSE yielded a markedly sharper PSF for single‐point data. Figure 4b shows the simulated field map and circular phantom used to evaluate the impact of off‐resonance effects, together with quantitative comparisons using a gradient similarity metric. Across all tested off‐resonance levels, CORPUSE consistently achieved higher gradient similarity than conventional reconstruction, indicating improved preservation of structural boundaries (Figure 4c). Representative reconstructed phantom images under increasing field inhomogeneity are shown in Figures S3 and S4. Compared with conventional PROPELLER‐EPI reconstruction, CORPUSE substantially reduced off‐resonance–related artifacts and preserved much sharper object boundaries at higher B0 inhomogeneity levels. This improved edge definition is further supported by the 2D phantom reconstructions (Figure S3) and corresponding 1D intensity profiles (Figure S4), which consistently show steeper boundary transitions for CORPUSE across different off‐resonance conditions. Figure 4c further demonstrates the robustness of off‐resonance correction in CORPUSE under varying field inhomogeneities (yellow arrows) induced by different shimming settings. Notably, the CORPUSE‐reconstructed data showed clearer boundaries and better preservation of fine structural details (red arrows). Together, these simulation and phantom results confirm that CORPUSE maintains superior structural sharpness and geometric fidelity under off‐resonance conditions.

### 4.2 Assessment of Prior Constraints

**FIGURE 5:**
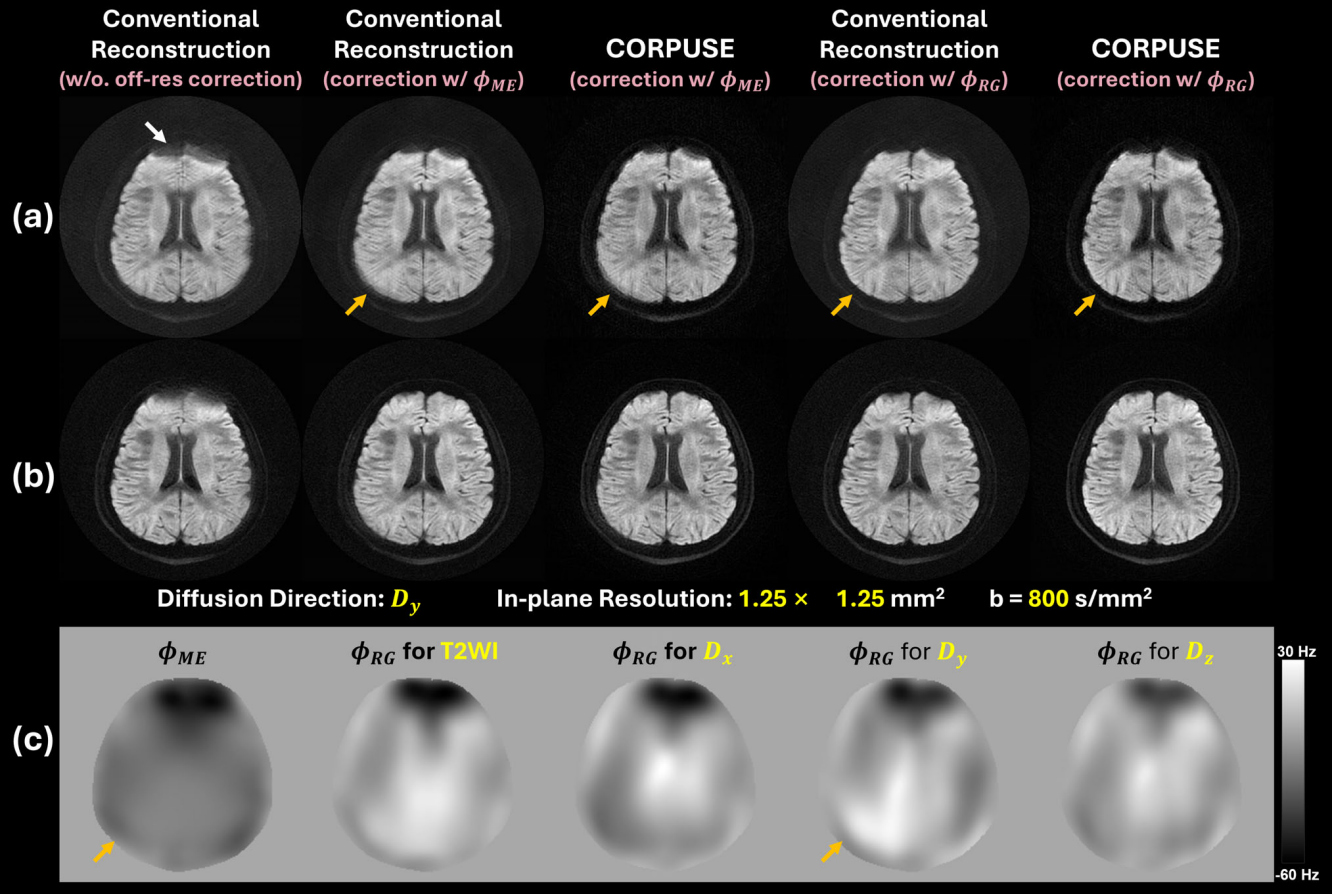
Comparison of off‐resonance correction using two types of field maps for DW‐PROPELLER‐EPI data. Reconstructed DW images for (a) 6‐blade and (b) 18‐blade acquisitions are shown for conventional PROPELLER‐EPI reconstruction (no correction, correction based on $\phi_{\mathrm{ME}}$, and correction based on $\phi_{\mathrm{RG}}$) and the CORPUSE framework (corrections base on either $\phi_{\mathrm{ME}}$ or $\phi_{\mathrm{RG}}$). Baseline DW images reconstructed using the conventional method without off‐resonance correction exhibit severe blurring, particularly in the frontal region (white arrow). For the 6‐blade data, $\phi_{\mathrm{RG}}$ demonstrates superior reduction of off‐resonance effects, while residual artifacts persisted with $\phi_{\mathrm{ME}}$ (orange arrows). (c) Comparison of direction‐specific $\phi_{\mathrm{RG}}$ and ME‐GRE derived $\phi_{\mathrm{ME}}$ field maps across different diffusion direction, illustrating the improved off‐resonance correction achieved with $\phi_{\mathrm{RG}}$ relative to $\phi_{\mathrm{ME}}$.

Figure 5 compares off‐resonance correction using $\phi_{\mathrm{RG}}$ and $\phi_{\mathrm{ME}}$ field maps for six‐blade (Figure 5a) and 18‐blade (Figure 5b) DW‐PROPELLER‐EPI data, reconstructed using either conventional or CORPUSE framework. Baseline DW images reconstructed using the conventional method without off‐resonance correction exhibit severe blurring caused by blade‐specific distortion, particularly, in the frontal region (white arrow). Both reconstruction methods substantially reduced blurring when either $\phi_{\mathrm{RG}}$ or $\phi_{\mathrm{ME}}$ was used for off‐resonance correction. For the six‐blade data, $\phi_{\mathrm{RG}}$ demonstrates superior reduction of off‐resonance effects, while residual artifacts persisted with $\phi_{\mathrm{ME}}$ (orange arrows). This suggests that $\phi_{\mathrm{RG}}$ can better account for direction‐specific distortion due to eddy current effects (Figure 5c). For the 18‐blade data, both field maps yielded comparable quality for DW images (Figure 5b).

**FIGURE 6:**
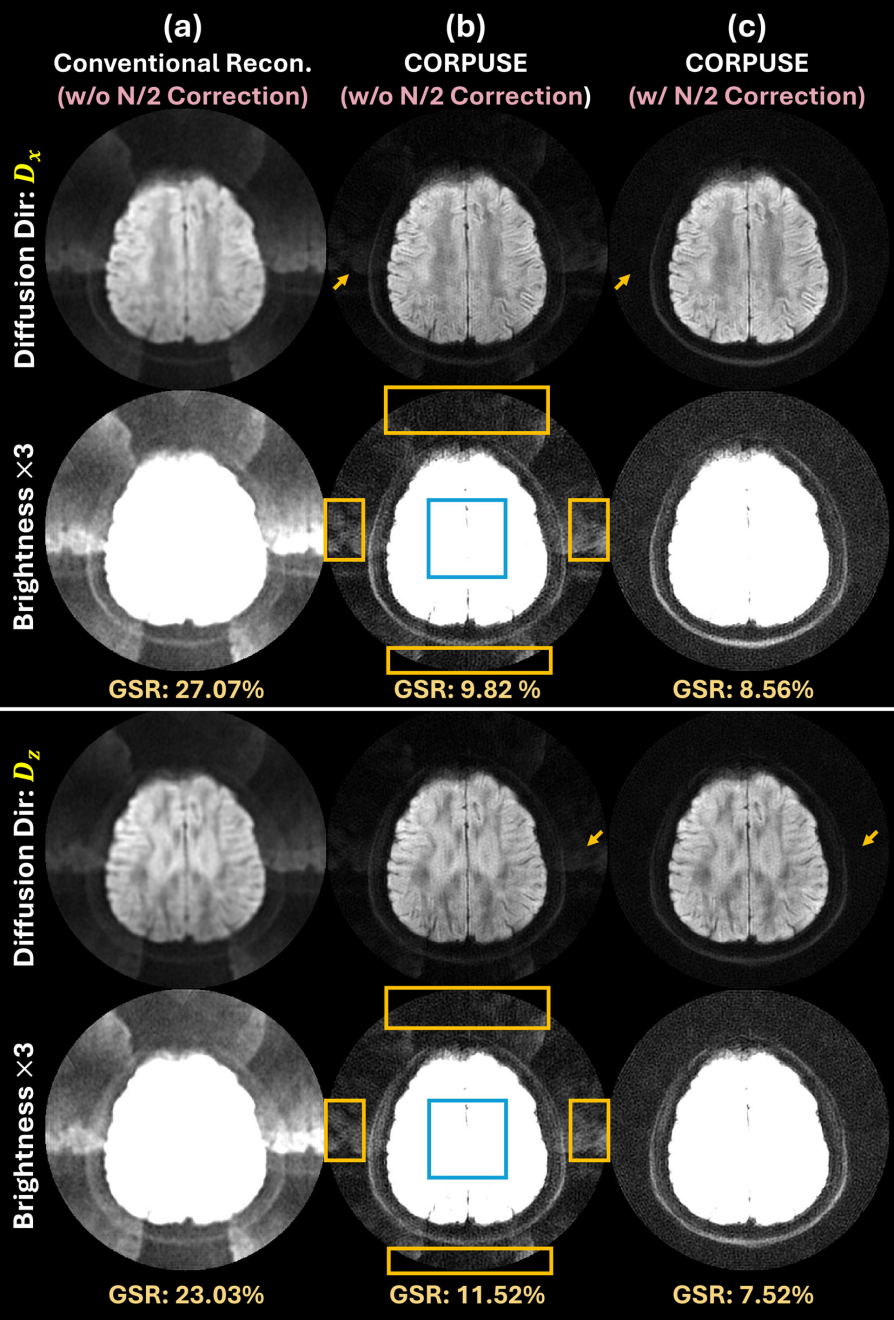
Comparison of Nyquist ghost artifact suppression in 6‐blade DW‐PROPELLER‐EPI data. Representative images from two diffusion directions are shown for (a) conventional PROPELLER‐EPI reconstruction without per‐blade Nyquist ghost correction, (b) CORPUSE without the 2D Nyquist ghost phase error constraint, and (c) CORPUSE with the 2D Nyquist ghost phase error constraint. Note that blurring in the frontal region, visible in all reconstructed images, reflects the intentional exclusion of off‐resonance correction to isolate the effect of ghost suppression in this comparison. Conventional PROPELLER‐EPI reconstruction without per‐blade Nyquist ghost correction exhibited pronounced background ghost artifacts. The CORPUSE framework significantly reduced these ghosts even without explicit ghost correction. Full incorporation of the 2D Nyquist ghost phase errors into the iterative CORPUSE framework yielded the most effective artifact suppression.

Figure 6 demonstrates the efficacy of the CORPUSE framework in suppressing Nyquist ghost artifacts for the LAP‐EPI data acquired with two different diffusion directions. Without Nyquist ghost correction applied to each blade, the conventional PROPELLER‐EPI reconstruction exhibits pronounced ghosting artifacts spreading across the image background with a high GSR (Figure 6a). In contrast, even without explicitly correcting the 2D Nyquist ghost phase errors, the CORPUSE framework significantly reduced these background ghosts (Figure 6b). Most notably, when the 2D Nyquist ghost phase errors were incorporated into the iterative process, CORPUSE further suppressed the Nyquist ghost artifact in each blade (Figure 6c).

### 4.3 CORPUSE Reconstruction Under Different Scan Parameters

**FIGURE 7:**
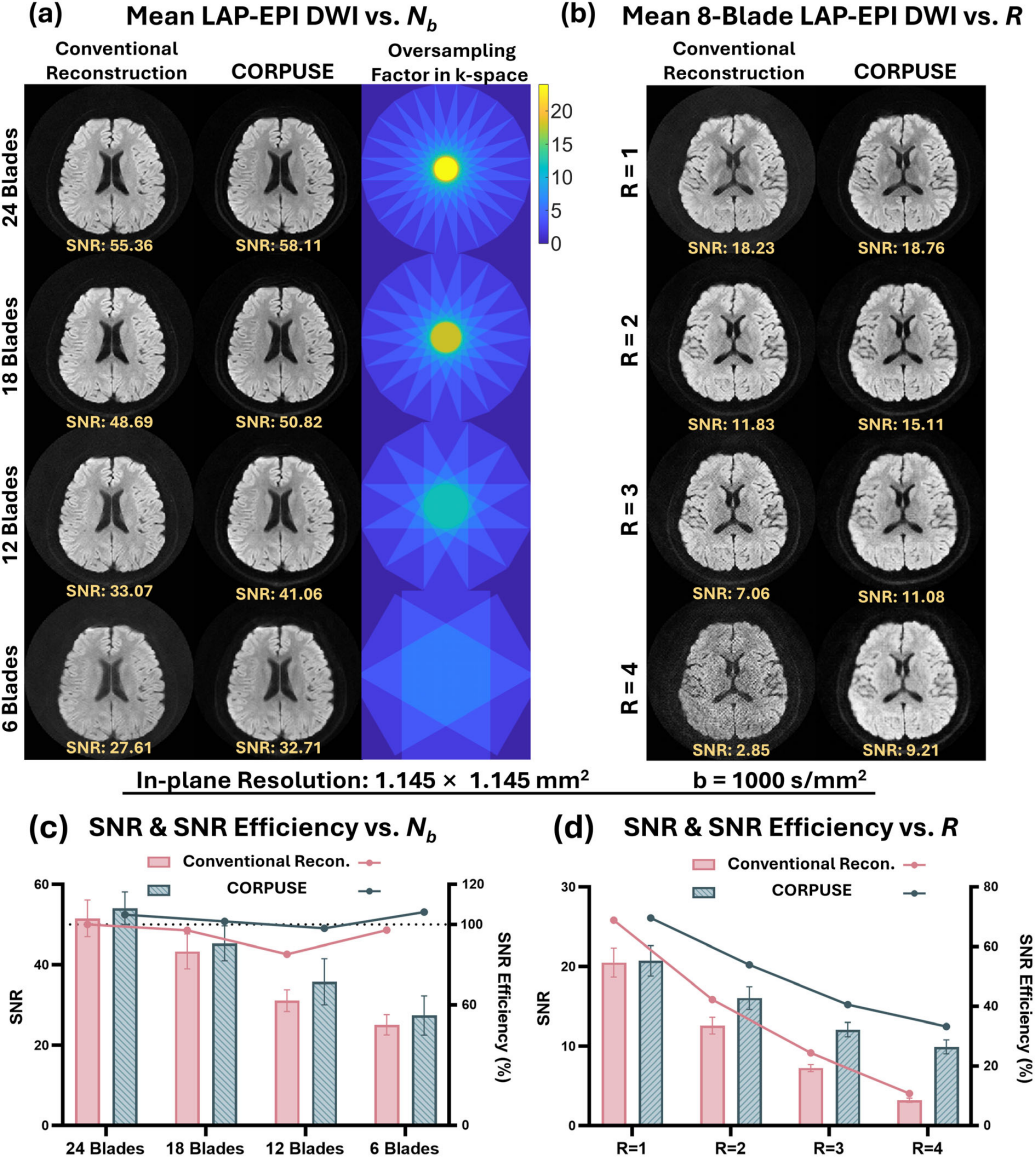
Comparison of reconstruction performance between conventional PROPELLER‐EPI reconstruction and the CORPUSE framework for LAP‐EPI data under varying acquisition parameters. Visual comparisons across (a) different numbers of blades ($N_{b}$= 24 to 6) for non‐accelerated LAP‐EPI data and (b) different per‐blade acceleration factors (*R* = 1 to 4) for 8‐blade LAP‐EPI data. Quantitative comparisons of SNR and SNR efficiency are shown for (c) varying numbers of blades and (d) per‐blade acceleration factors. CORPUSE demonstrated modest SNR efficiency improvements for non‐accelerated data, while achieving significantly higher performance across all accelerated scenarios compared to conventional PROPELLER‐EPI reconstruction.

Figure 7 summarizes the visual and quantitative comparisons between conventional PROPELLER‐EPI reconstruction and the proposed CORPUSE framework across varying scan parameters. In Figure 7a, reducing the number of blades (24–6) lowered SNR for both methods due to decreased oversampling, yet CORPUSE consistently yielded lower noise and improved DW image quality, with an average SNR improvement of 9% across unaccelerated datasets. Figure 7b further demonstrates the advantage of CORPUSE under per‐blade acceleration. While conventional PROPELLER‐EPI reconstruction exhibited increasingly severe noise amplification as *R* increased from 1 to 4, CORPUSE effectively suppressed this trend and maintained stable image quality. Quantitatively, CORPUSE improved SNR efficiency by 1% (*R* = 1), 28% (*R* = 2), 67% (*R* = 3), and up to 209% (*R* = 4) relative to conventional PROPELLER‐EPI reconstruction. These improvements were consistent across all 22 slice locations (Table S2), and full SNR maps for all datasets are shown in Figures S5 and S6.

**FIGURE 8:**
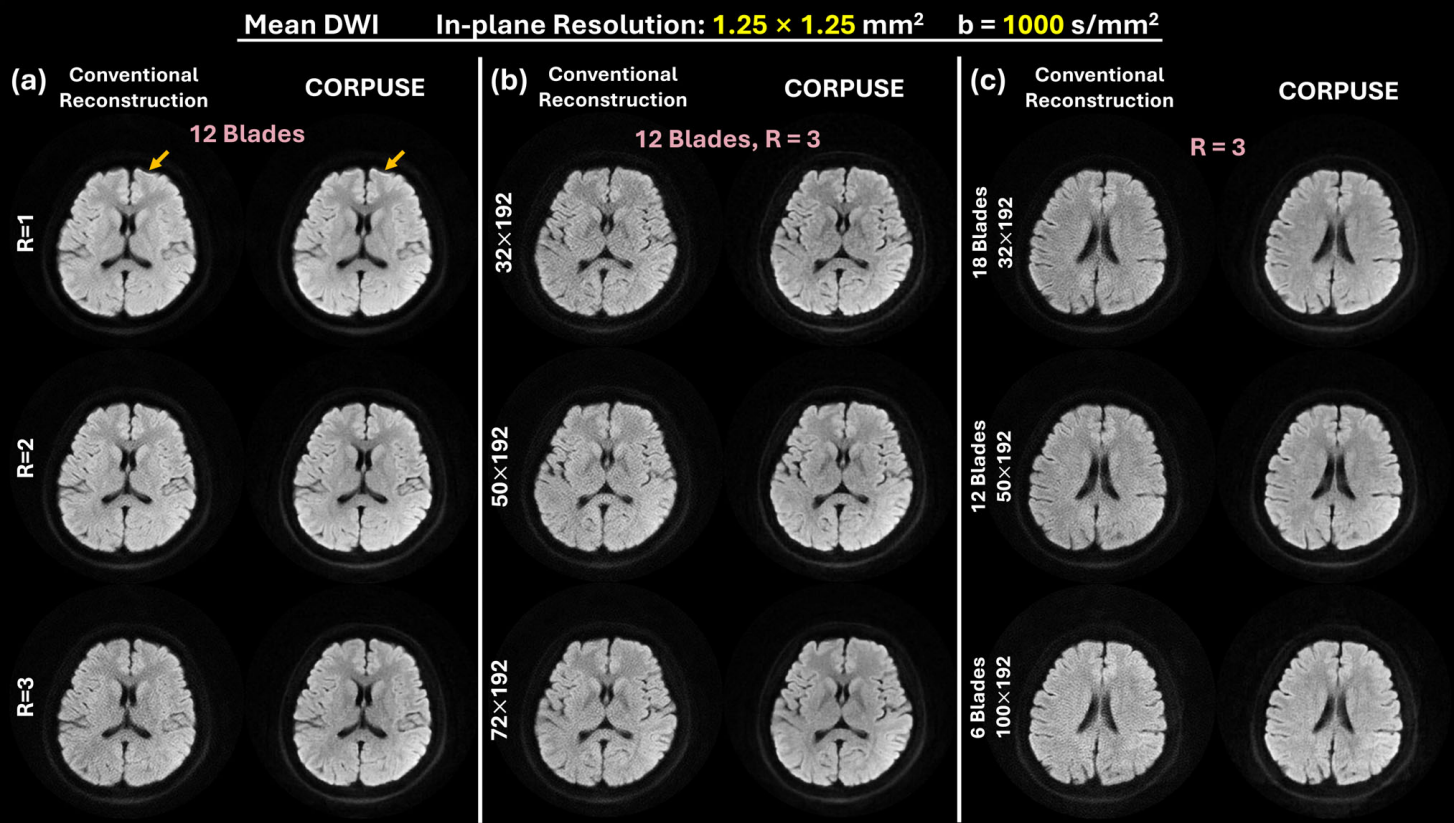
Comparison of conventional PROPELLER‐EPI reconstruction and CORPUSE under different SAP‐EPI acquisition configurations. Mean diffusion‐weighted images are shown. (a) Effect of parallel imaging acceleration (*R* = 1–3) for a 12‐blade acquisition (blade width = 50 × 192). At *R* = 1, both reconstructions exhibit residual distortion near regions with strong off‐resonance effects (orange arrows), whereas higher per‐blade acceleration improves geometric fidelity. (b) Effect of blade width (32 × 192, 50 × 192, and 72 × 192) for a 12‐blade acquisition with *R* = 3. CORPUSE better preserves structural detail and suppresses noise amplification compared with conventional reconstruction. (c) Effect of the number of blades (18, 12, and 6 blades) with *R* = 3 for a 360° k‐space coverage. Across all configurations, CORPUSE reconstruction consistently improves image sharpness and reduces noise amplification compared with conventional reconstruction, particularly at higher per‐blade acceleration and with fewer blades.

Figure 8 compares SAP‐EPI reconstructions across different acquisition configurations. For a 12‐blade acquisition with varying per‐blade acceleration (*R* = 1–3), Figure 8a demonstrates that both conventional PROPELLER‐EPI reconstruction and CORPUSE exhibited residual distortion near regions with strong off‐resonance effects at *R* = 1 (orange arrows). This is consistent with the increased sensitivity to off‐resonance and T2* decay associated with the longer ETL in SAP‐EPI without per‐blade acceleration. In this setting, field map correction alone could not fully recover regions with severe distortion, whereas applying per‐blade acceleration further improved geometric fidelity. Figure 8b demonstrates that when blade width was varied at a constant per‐blade acceleration (*R* = 3), CORPUSE preserved structural detail and suppressed noise amplification relative to conventional reconstruction. Similarly in Figure 8c, when the number of blades was reduced, conventional reconstruction showed increased noise amplification and image degradation, whereas CORPUSE maintained stable reconstruction quality. This observation is consistent with the quantitative SNR measurements reported in Table S2 for the LAP‐EPI experiments. Overall, these results demonstrate that CORPUSE provides robust and consistent SAP‐EPI reconstruction across a wide range of acquisition parameters.

**FIGURE 9:**
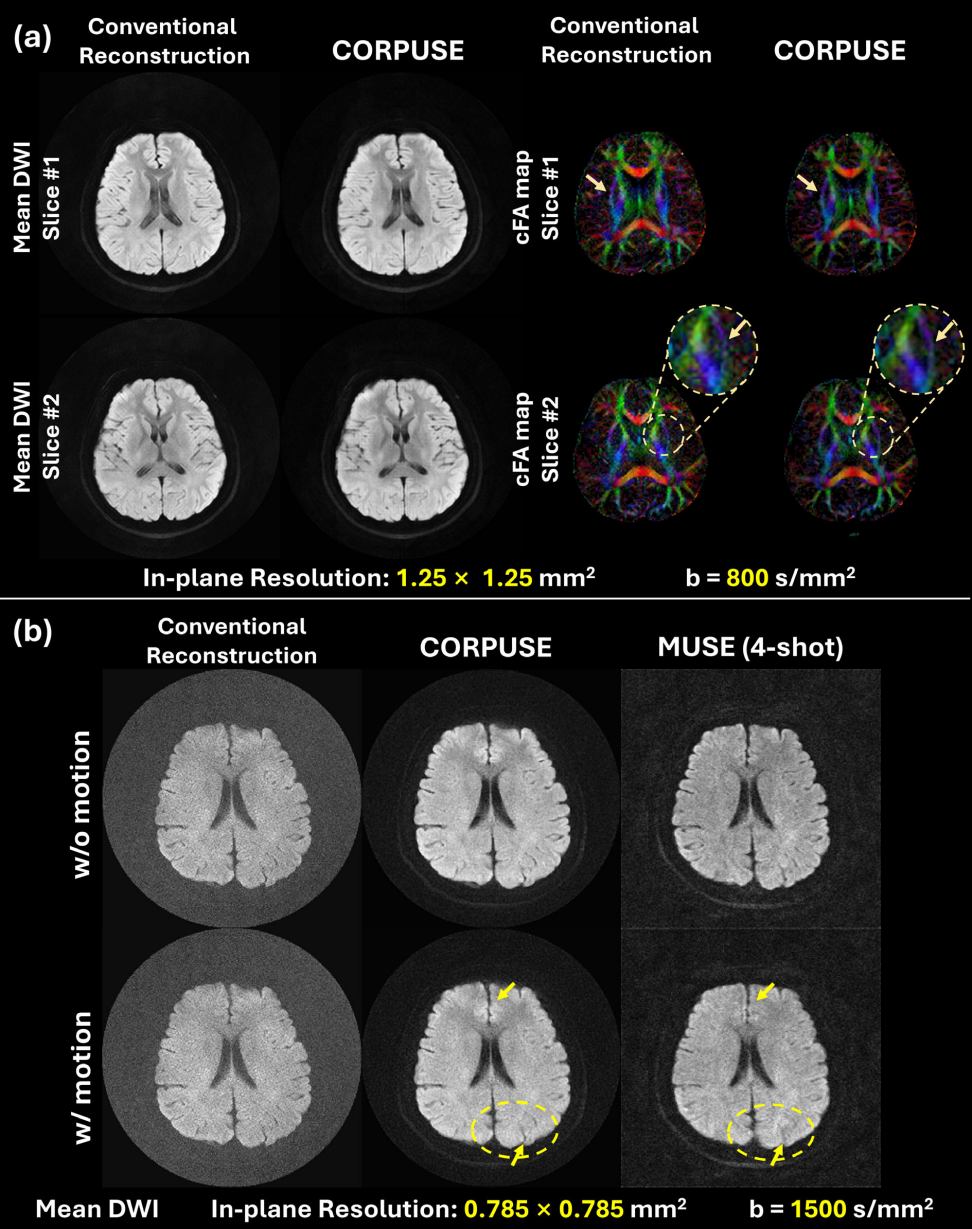
(a) DTI feasibility comparison using 8‐blade LAP‐EPI data acquired with 15 diffusion directions (*R* = 2). Mean DW images and cFA maps were computed from datasets reconstructed with either conventional PROPELLER‐EPI reconstruction or the CORPUSE framework. cFA maps derived from the CORPUSE‐reconstructed data exhibited superior noise suppression and improved delineation of white matter tracts, with noticeably sharper structural boundaries compared to those produced by conventional PROPELLER‐EPI reconstruction, particularly evident in the zoomed regions. (b) Comparison between conventional PROPELLER‐EPI reconstruction, CORPUSE framework, and four‐shot MUSE DWI under conditions with and without subtle head motion. CORPUSE yields sharper structural detail and reduced noise compared to conventional PROPELLER‐EPI reconstruction, while maintaining robustness to subtle motion and preserving fine anatomical features (yellow arrows). In contrast, four‐shot MUSE DWI exhibits pronounced motion‐induced blurring and diminished signal fidelity.

Figure 9a presents representative mean DW images and cFA maps from a 15‐direction DTI dataset (eight‐blade LAP‐EPI, *R* = 2). CORPUSE produced visibly cleaner cFA maps with enhanced delineation of white matter tracts and sharper anatomical boundaries, as highlighted in the zoomed regions. Figure 9b compares the conventional PROPELLER‐EPI, the CORPUSE framework, and time‐matched four‐shot MUSE DWI under high *b*‐value and high‐resolution conditions. In the motion‐free comparison (upper row), the CORPUSE reconstruction of the 24‐blade LAP‐EPI data demonstrated superior noise suppression compared to both conventional PROPELLER‐EPI reconstruction and MUSE DWI. With subtle head motion (bottom row), CORPUSE retained the intrinsic motion robustness of PROPELLER‐EPI while substantially reducing noise, whereas four‐shot MUSE DWI exhibited motion‐induced blurring. As indicated by the yellow arrows, CORPUSE effectively preserved fine structural details and minimized motion‐induced blurring.

## 5 Discussion

PROPELLER‐EPI is a multi‐shot technique that uses intrinsic self‐navigation to achieve high‐quality DWI, but its performance is limited by long scan times from *k*‐space oversampling and the need for per‐blade acceleration paired with off‐resonance correction to prevent blurring following blade combination. The proposed CORPUSE framework enables collaborative multiblade reconstruction through an iterative process incorporating multiple constraints to achieve: (1) simultaneous correction of interblade phase variations and 2D Nyquist ghost phase errors and (2) enhanced geometric fidelity. These constraints preserve image quality and minimize SNR loss even when the number of blades is reduced to shorten scan time, while enabling higher per‐blade acceleration with a reduced SNR penalty compared to conventional PI. Notably, CORPUSE is compatible with both LAP‐EPI and SAP‐EPI trajectories.

Previous iterative reconstruction for PROPELLER acquisitions have often neglected EPI‐related phase errors and off‐resonance effects [22, 23, 24, 25, 26]. CORPUSE addresses these by incorporating composite 2D phase errors (Nyquist ghost phase errors and inter‐blade phase variations) and an off‐resonance correction operator within the POCSMUSE framework. Leveraging the self‐referenced nature of PROPELLER‐EPI, blade‐specific 2D phase errors and field maps (estimated from blade pair with opposite PE polarity) can be obtained without additional calibration scans. Since each blade exhibits unique distortion along the PE direction, using coil sensitivity profiles without distortion matching can bias phase estimation and induce residual artifacts in regions with severe off‐resonance (Figure S7). CORPUSE therefore utilizes blade‐specific distortion‐matched coil sensitivity profiles during both phase error estimation and iterative reconstruction. Additionally, beyond the explicit modeling of off‐resonance and phase errors, the iterative collaborative reconstruction in CORPUSE may further suppress residual inconsistencies across blades, thereby helping reduce artifacts such as 2D Nyquist ghosts (Figure 6).

Off‐resonance correction remains essential for both conventional PROPELLER‐EPI reconstruction and CORPUSE to reduce blurring. Although in vivo experiments were performed on a 1.5T MRI scanner, simulations and phantom experiments with synthetically amplified field inhomogeneity demonstrated that CORPUSE remains robust under severe off‐resonance conditions. In vivo results showed sharper structural detail with CORPUSE than with conventional reconstruction (Figure 4). Because CORPUSE better preserves high‐frequency information, Gibbs ringing from truncated *k*‐space sampling may become more visible near sharp boundaries. Such ringing arises intrinsically from truncated *k*‐space sampling and could be mitigated using classical Gibbs suppression techniques [44] or emerging AI‐based deringing approaches if necessary [45].

Regarding the field map source, the RG‐based field map generally provided more effective distortion correction than the ME‐GRE‐derived field map, with the largest advantage observed in the six‐blade DW‐PROPELLER‐EPI data (Figure 5a). This likely reflects that the RG‐based field map derived directly from diffusion‐weighted opposing blade pairs can capture eddy‐current‐induced distortion components associated with diffusion gradients that a separately acquired ME‐GRE field map cannot measure. However, this advantage diminished as angular sampling density increased (18 blades in Figure 5b), indicating that oversampling partially suppresses residual distortion. Additionally, RG‐based field map estimation requires sufficient consistency between opposing blades and may therefore be more vulnerable to interblade motion. In the presence of interblade motion, an independently acquired ME‐GRE field map may provide a more robust alternative. Furthermore, our comparison between a single RG‐based field map estimated from one opposing blade pair and multiple blade‐specific RG‐based field maps estimated from all available opposing pairs showed only modest differences in reconstruction quality, with the benefit of blade‐specific field maps becoming more visible mainly in a fewer blade setting (such as six‐blade data shown in Figure S2). Taken together, these findings suggest that direction‐specific RG‐based field maps are most beneficial when fewer blades are used because residual distortion is then less effectively suppressed by oversampling. In contrast, for the acquisition settings used in this study, a single opposing‐pair estimate remains an effective and computationally efficient choice.

Despite its oversampling advantage, PROPELLER‐EPI typically suffers from low SNR efficiency, which is further degraded by per‐blade acceleration. CORPUSE substantially improved both SNR and SNR efficiency (Figure 7c,d), maintaining image quality with fewer blades and mitigating the parallel‐imaging penalty associated with per‐blade acceleration (Figure 7b,d). This improvement is particularly beneficial for wider‐blade LAP‐EPI acquisitions, which allow fewer blades, and for SAP‐EPI acquisitions, where long ETL can be mitigated through higher per‐blade acceleration while preserving geometric accuracy (Figures 7b and 8). In practice, the choice between LAP‐EPI and SAP‐EPI depends on the trade‐off among distortion sensitivity and scan efficiency. SAP‐EPI combined with CORPUSE is advantageous for noise suppression when higher acceleration or shorter scan time is desired, whereas LAP‐EPI benefits from wider blade coverage and greater *k*‐space redundancy, providing higher intrinsic SNR efficiency and improved reconstruction robustness. These performance gains translated into cleaner computed cFA maps with improved delineation of white matter tract (Figure 9a). CORPUSE improves the clinical trade‐off between scan time, SNR, and image quality for accelerated PROPELLER‐EPI, enabling shorter scans without compromising diagnostic detail.

Additionally, prior studies on ms‐EPI‐DWI reconstruction have shown that the accuracy of phase‐correction information is limited not only by its intrinsic noise level but also by geometric mismatch between the auxiliary phase data and the imaging echo. This degradation can measurably affect the final reconstruction [46]. In CORPUSE, both the phase map and the field map are incorporated directly into the reconstruction pipeline, and thus the quality of these map estimations may influence the final image quality. Consistent with this, our supplementary retrospective parameter experiments with different blade‐width settings (Figures S8–S10) suggest that narrower blade widths can affect the stability of phase map estimation in CORPUSE, primarily contributing to a measurable, monotonic effect on the final reconstruction. In contrast, the isolated contribution of field‐map variation appears comparatively limited over the tested blade‐width range. Therefore, adequate blade width remains important not only for sampling efficiency but also for maintaining sufficiently reliable phase map estimation to ensure stable CORPUSE reconstruction.

Among ms‐EPI techniques, PROPELLER‐EPI exhibits superior motion robustness. As shown in Figure 9b, PROPELLER‐EPI reconstructed with CORPUSE exhibits improved motion resilience compared with MUSE DWI. While MUSE relies on explicit phase estimation and intershot alignment that remain sensitive to motion, the overlapping central *k*‐space in PROPELLER‐EPI stabilizes blade‐to‐blade inconsistencies. CORPUSE leverages this redundancy to enable high‐resolution, high‐b‐value DWI with enhanced motion resilience. This self‐navigated robustness also distinguishes PROPELLER‐EPI from other ms‐EPI approaches, such as readout‐segmented EPI (rs‐EPI) [47], which requires additional navigators and is more motion sensitive.

This study demonstrates the potential of CORPUSE to improve image quality and scan efficiency for PROPELLER‐EPI, while also revealing several limitations. First, accurate estimation of composite 2D phase errors remains constrained by PI performance, as accelerated sampling can introduce noise that biases phase estimation. Second, although CORPUSE improves scan efficiency, total acquisition time remains several‐fold longer than single‐shot EPI, posing challenges for routine clinical use. Further improvements may be achieved by combining CORPUSE with advanced PROPELLER trajectories (e.g., Turboprop [48], X‐PROP [49]), or simultaneous multislice (SMS) [50, 51, 52] techniques. Finally, the current implementation does not incorporate explicit rigid‐body motion correction and may therefore be less robust in the presence of substantial motion. In principle, the standard PROPELLER bulk motion correction strategy, based on blade‐wise estimation of rigid‐body rotations and translations from low‐resolution central *k*‐space, could be incorporated into CORPUSE as additional geometric operators in the forward model [53]. For diffusion imaging, the corresponding rotation estimates could also be used for *b*‐matrix correction by updating the diffusion gradient directions [54, 55].

## 6 Conclusion

In this work, we introduced CORPUSE, a collaborative multiblade, constraint‐driven reconstruction framework for DW‐PROPELLER‐EPI. By integrating corrections for 2D Nyquist‐ghost phase errors, interblade phase variations, and off‐resonance effects, CORPUSE enables high‐quality reconstruction even with fewer blades and under substantial per‐blade acceleration. This capability substantially improves scan efficiency while alleviating the off‐resonance and T2*‐decay penalties that arise when wider blades and longer ETL are used to reduce the total number of blades required for gapless *k*‐space coverage. Importantly, CORPUSE preserves the intrinsic motion robustness of PROPELLER‐EPI while reducing noise amplification and maintaining image fidelity in accelerated acquisitions.

## Funding

This work was supported by Research Grants Council (RGC) of Hong Kong, ECS24213522, GRF14206723, GRF14213225, GRF17106820, GRF17125321 and Innovation and Technology Commission (ITC), The Government of Hong Kong Special Administrative Region.

## Supporting information


**Figure S1:** Trade‐offs in the number of blade ($N_{b}$) for a target reconstruction matrix size (e.g., 192 × 192).
**Figure S2:** Comparison of CORPUSE reconstructions using two RG‐based field map estimation strategies.
**Figure S3:** Reconstruction results of the circular phantom under simulated B0 inhomogeneity levels of 0, 50, 150, and 300 Hz.
**Figure S4:** One‐dimensional intensity profiles extracted from the simulated circular phantom under varying off‐resonance conditions.
**Figure S5:** The comparison of SNR maps between conventional PROPELLER‐EPI reconstruction (a, c, e, g) and the CORPUSE framework (b, d, f, h) for non‐accelerated LAP‐EPI data under different numbers of blades ($N_{b}$=24–6).
**Figure S6:** The comparison of SNR maps between conventional PROPELLER‐EPI reconstruction (a, c, e, g) and the CORPUSE framework (b, d, f, h) for eight‐blade LAP‐EPI data under different per‐blade acceleration factors (*R* = 1–4).
**Figure S7:** Effect of distortion matching in coil sensitivity profiles for CORPUSE reconstruction.
**Figure S8:** Parameter experiment evaluating the effect of phase term estimation from different blade widths, while keeping the field map fixed to that estimated from the reference 192 × 50 blades.
**Figure S9:** Parameter experiment evaluating the effect of field map estimation from different blade widths, while keeping the phase map fixed to that estimated from the reference 192 × 50 blades.
**Figure S10:** Parameter experiment evaluating the combined effect of phase map and field map estimation from different blade widths.

**Table S1:** Primary scan parameters for Short‐Axis DW‐PROPELLER‐EPI acquisitions.
**Table S2:** SNR and SNR efficiency comparison of the PROPELLER‐EPI method and proposed CORPUSE method.

## Data Availability

The data that support the findings of this study are available from the corresponding author upon reasonable request.
