# Supplementary material for: Collaborative Reconstruction of PROPELLER‐EPI Data Using POCSMUSE (CORPUSE) for High‐Fidelity Diffusion MRI
Source: Magn Reson Med. 2026 May 30;96(4):1929–46. doi: 10.1002/mrm.70448 (PMC13421002; doi:10.1002/mrm.70448)
Supplement: Supplementary file 1 — Figure S1: Trade‐offs in the number of blade (Nb) for a target reconstruction matrix size (e.g., 192 × 192). Figure S2: Comparison of CORPUSE reconstructions using two RG‐based field map estimation strategies. Figure S3: Reconstruction results of the circular phantom under simulated B0 inhomogeneity levels of 0, 50, 150, and 300 Hz. Figure S4: One‐dimensional intensity profiles extracted from the simulated circular phantom under varying off‐resonance conditions. Figure S5: The comparison of SNR maps between conventional PROPELLER‐EPI reconstruction (a, c, e, g) and the CORPUSE framework (b, d, f, h) for non‐accelerated LAP‐EPI data under different numbers of blades (Nb=24–6). Figure S6: The comparison of SNR maps between conventional PROPELLER‐EPI reconstruction (a, c, e, g) and the CORPUSE framework (b, d, f, h) for eight‐blade LAP‐EPI data under different per‐blade acceleration factors (R = 1–4). Figure S7: Effect of distortion matching in coil sensitivity profiles for CORPUSE reconstruction. Figure S8: Parameter experiment evaluating the effect of phase term estimation from different blade widths, while keeping the field map fixed to that estimated from the reference 192 × 50 blades. Figure S9: Parameter experiment evaluating the effect of field map estimation from different blade widths, while keeping the phase map fixed to that estimated from the reference 192 × 50 blades. Figure S10: Parameter experiment evaluating the combined effect of phase map and field map estimation from different blade widths. [file MRM-96-1929-s002.docx]

**
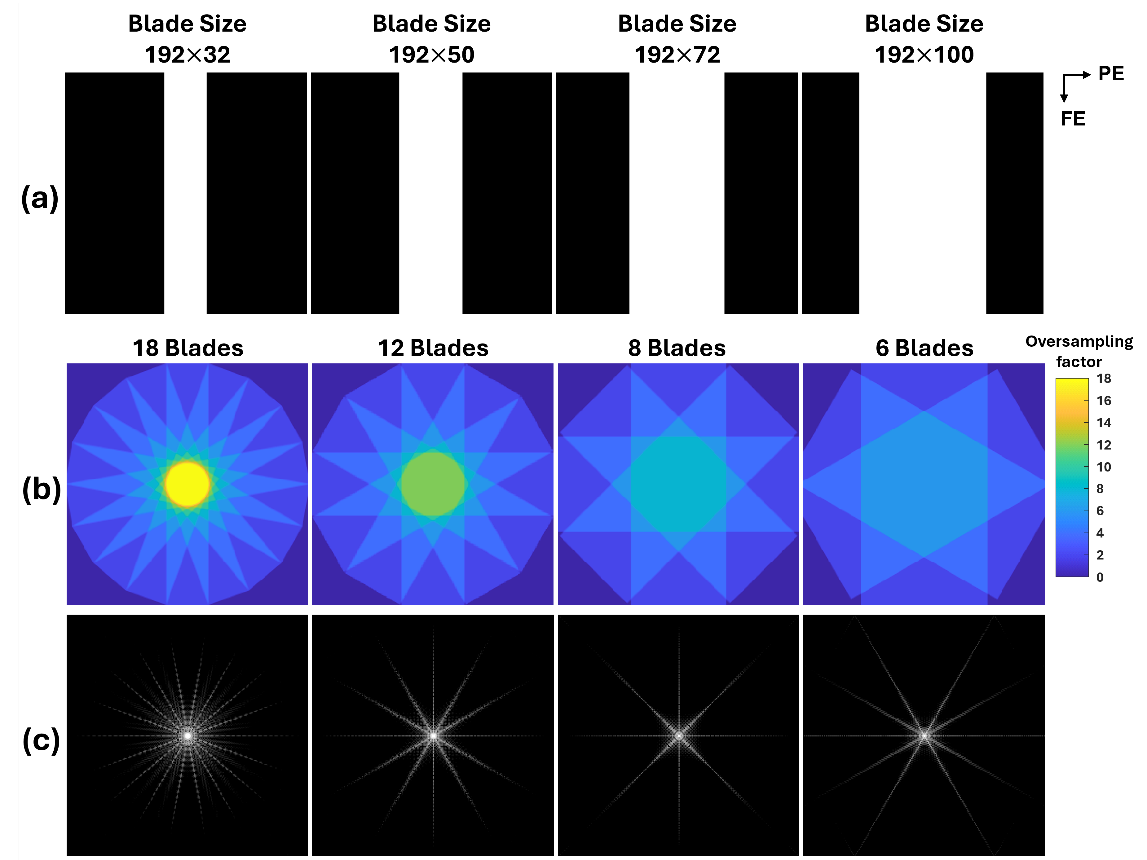
**

**Figure S1.** Trade-offs in the number of blade ($N_{b}$) for a target reconstruction matrix size (e.g., 192 $\times$ 192). (a) Reducing $N_{b}$for 360° k-space coverage requires increased angular coverage per blade (i.e., wider blades) to ensure gapless k-space sampling. (b) This results in a reduced k-space oversampling factor and decreased angular sampling density. As central k-space is critical for SNR, reduced oversampling in central region degrades both SNR and image quality. (c) Fewer blades (i.e., increased angular separation) also produce stronger radial streaking in the point spread function (PSF) (on a log scale), potentially compromising spatial resolution. In addition, wider blade necessitates a longer ETL, exacerbating T2* blurring and off-resonance artifacts.


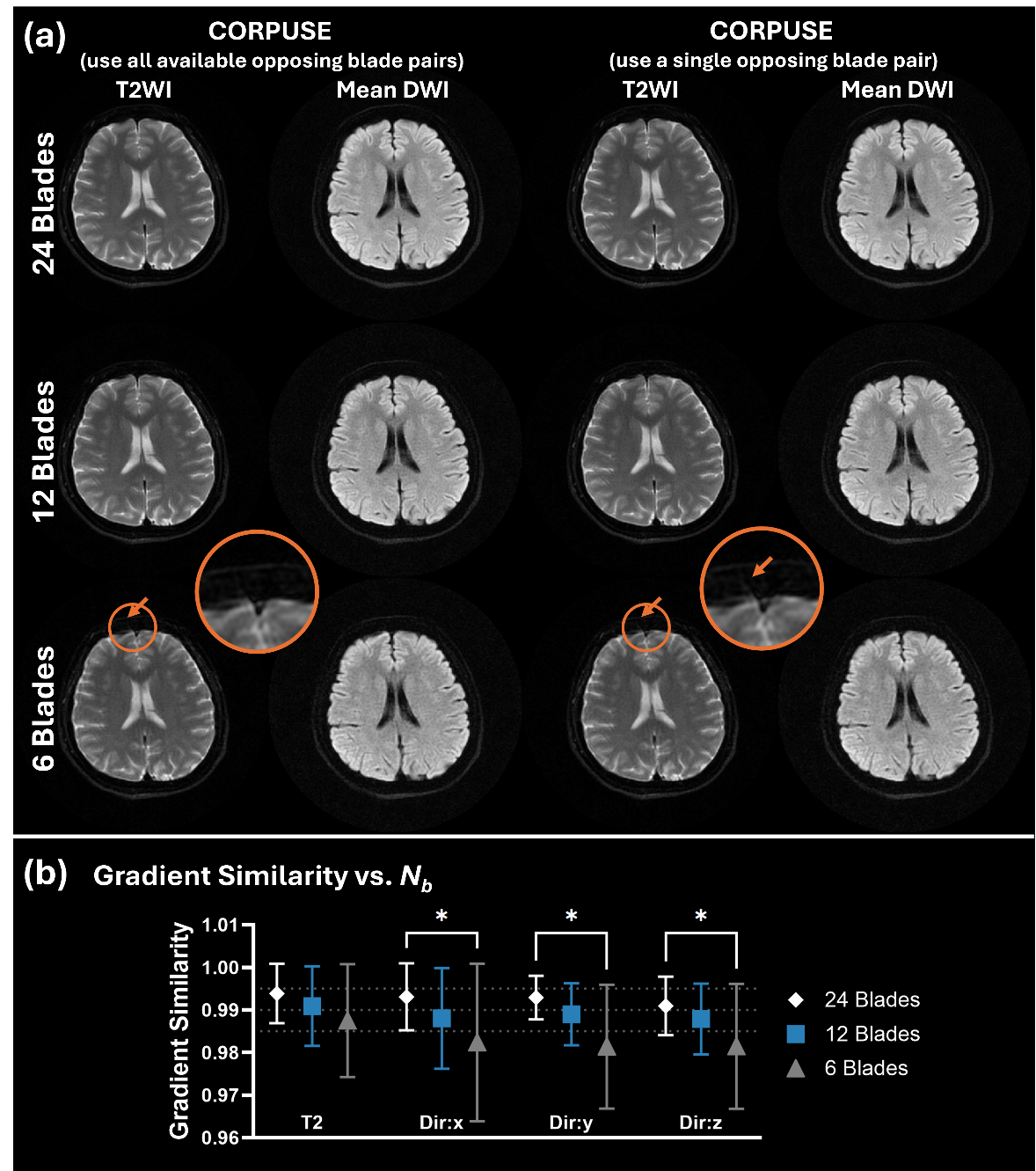


**Figure S2.** Comparison of CORPUSE reconstructions using two RG-based field map estimation strategies: (i) multiple blade-specific field maps estimated separately from all available opposing blade pairs, and (ii) a single RG-based field map estimated from one opposing blade pair (0° and 180°) and applied in reconstruction. For each blade-number setting (24, 12, and 6 blades), CORPUSE reconstruction was performed using both field map strategies. (a) Representative T2-weighted and diffusion-weighted reconstructions for DW-PROPELLER-EPI datasets with 24, 12, and 6 blades. Arrows highlight regions where slight geometric differences are observed when fewer blades are used. (b) Quantitative comparison using a gradient-based similarity metric for T2-weighted images and three diffusion directions (Dir:x, Dir:y, Dir:z). Within each blade-number condition, gradient similarity was computed between reconstructions obtained with all-pair-based field maps and those obtained with a single-pair-based field map. A statistically significant difference between the 24-blade and 6-blade conditions is observed (*p < 0.001).

**
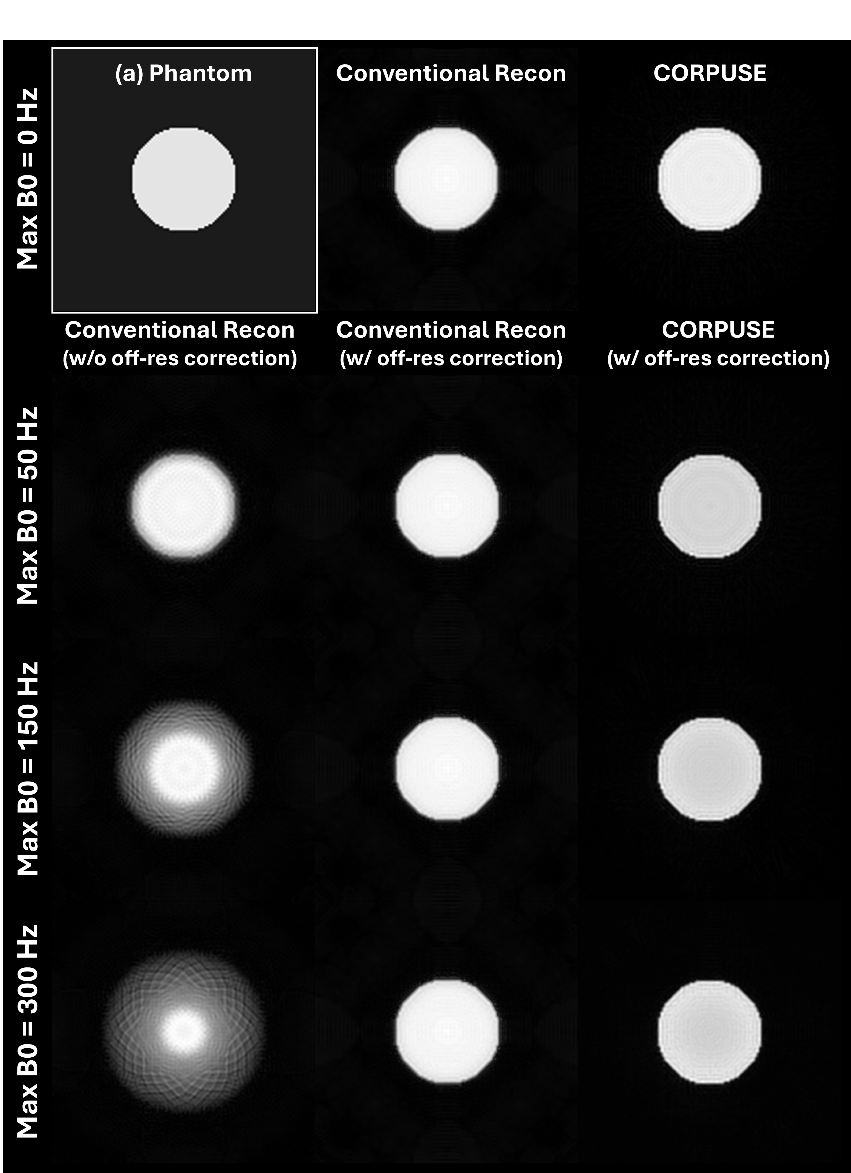
**

**Figure S3.** Reconstruction results of the circular phantom under simulated B0 inhomogeneity levels of 0, 50, 150, and 300 Hz. (a) shows the ground-truth phantom. For each inhomogeneity level, the middle columns display conventional PROPELLER-EPI reconstructions without and with off-resonance correction, while the rightmost column shows the corresponding CORPUSE results. Increasing B0 inhomogeneity leads to substantial blurring and geometric distortion in conventional reconstructions without correction, which can be mitigated by off-resonance correction. In contrast, CORPUSE maintains sharper boundaries and more accurate geometry across all field inhomogeneity conditions, demonstrating improved robustness to off-resonance effects.


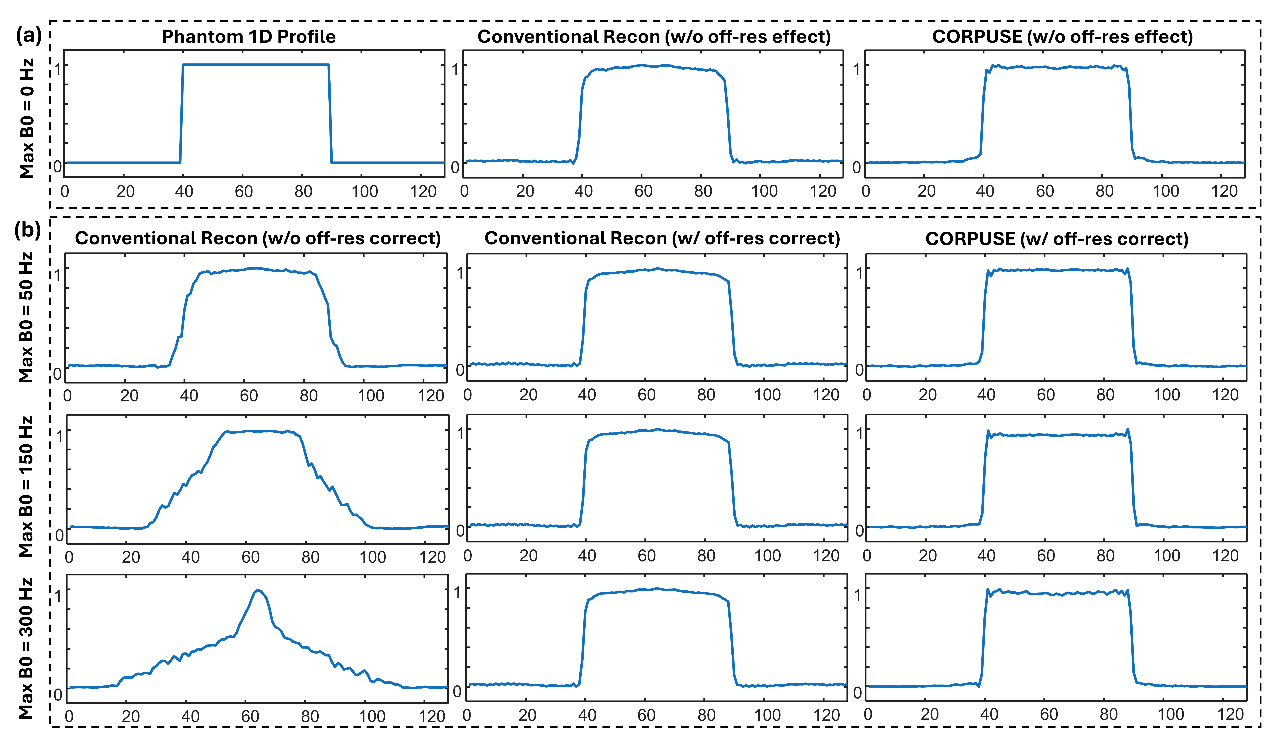


**Figure S4.** One-dimensional intensity profiles extracted from the simulated circular phantom under varying off-resonance conditions. (a) Ground-truth phantom profile and reconstructed profiles obtained without off-resonance effects using conventional PROPELLER-EPI reconstruction and CORPUSE. (b) Reconstructed profiles under increasing B0 inhomogeneity (50, 150, and 300 Hz). Columns show conventional PROPELLER-EPI reconstruction without off-resonance correction, conventional reconstruction with off-resonance correction, and CORPUSE reconstruction, respectively. Compared with conventional reconstruction, CORPUSE preserves steeper boundary transitions and better geometric fidelity across all off-resonance levels.


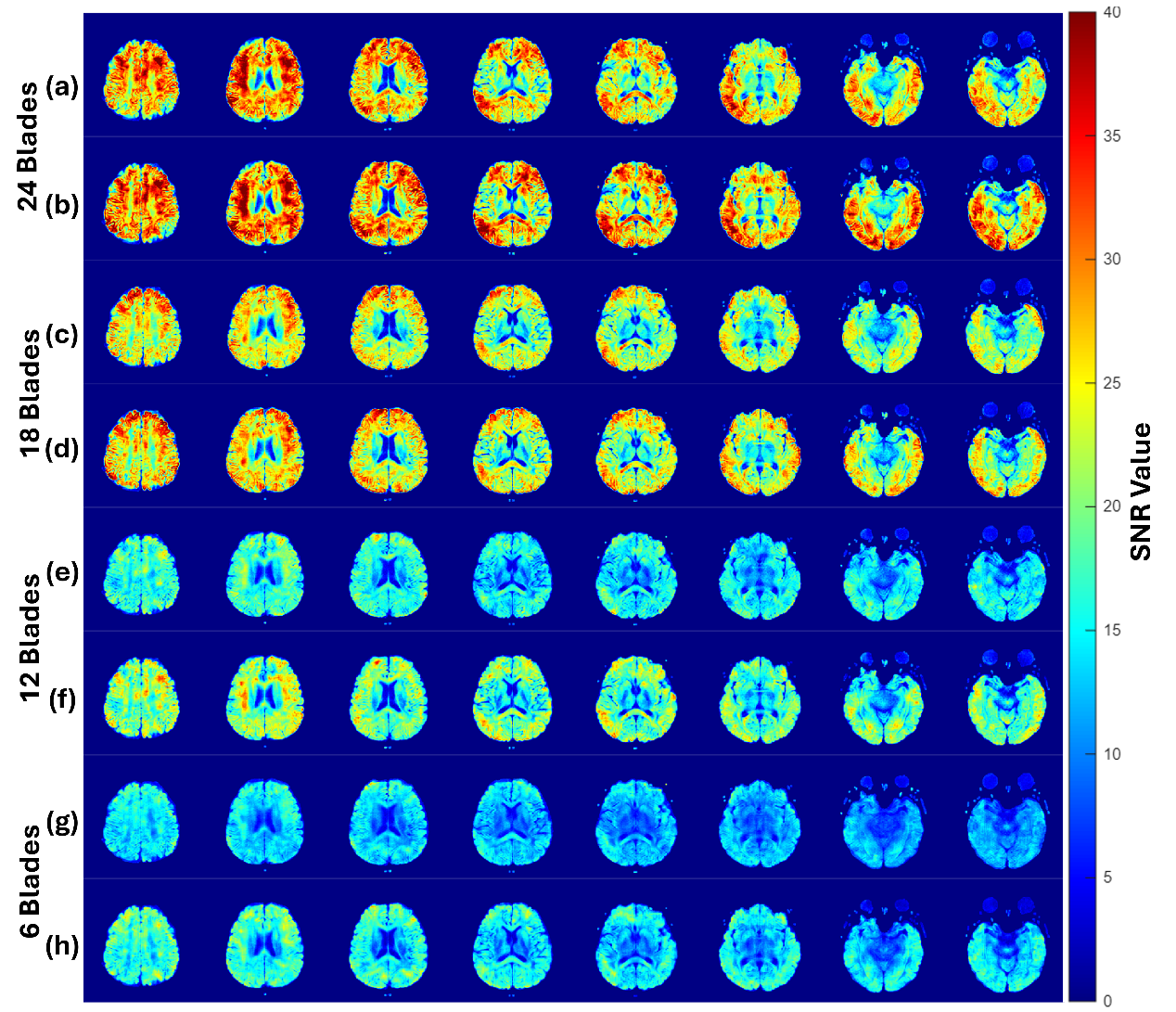


**Figure S5.** The comparison of SNR maps between conventional PROPELLER-EPI reconstruction (a, c, e, g) and the CORPUSE framework (b, d, f, h) for non-accelerated LAP-EPI data under different numbers of blades ($N_{b}$=24 to 6). Each column shows a matched slice location. While both methods yield comparable SNR at higher number of blades, CORPUSE provides higher SNR as the number of blades decreases, highlighting its advantage in reduced-blade acquisitions.


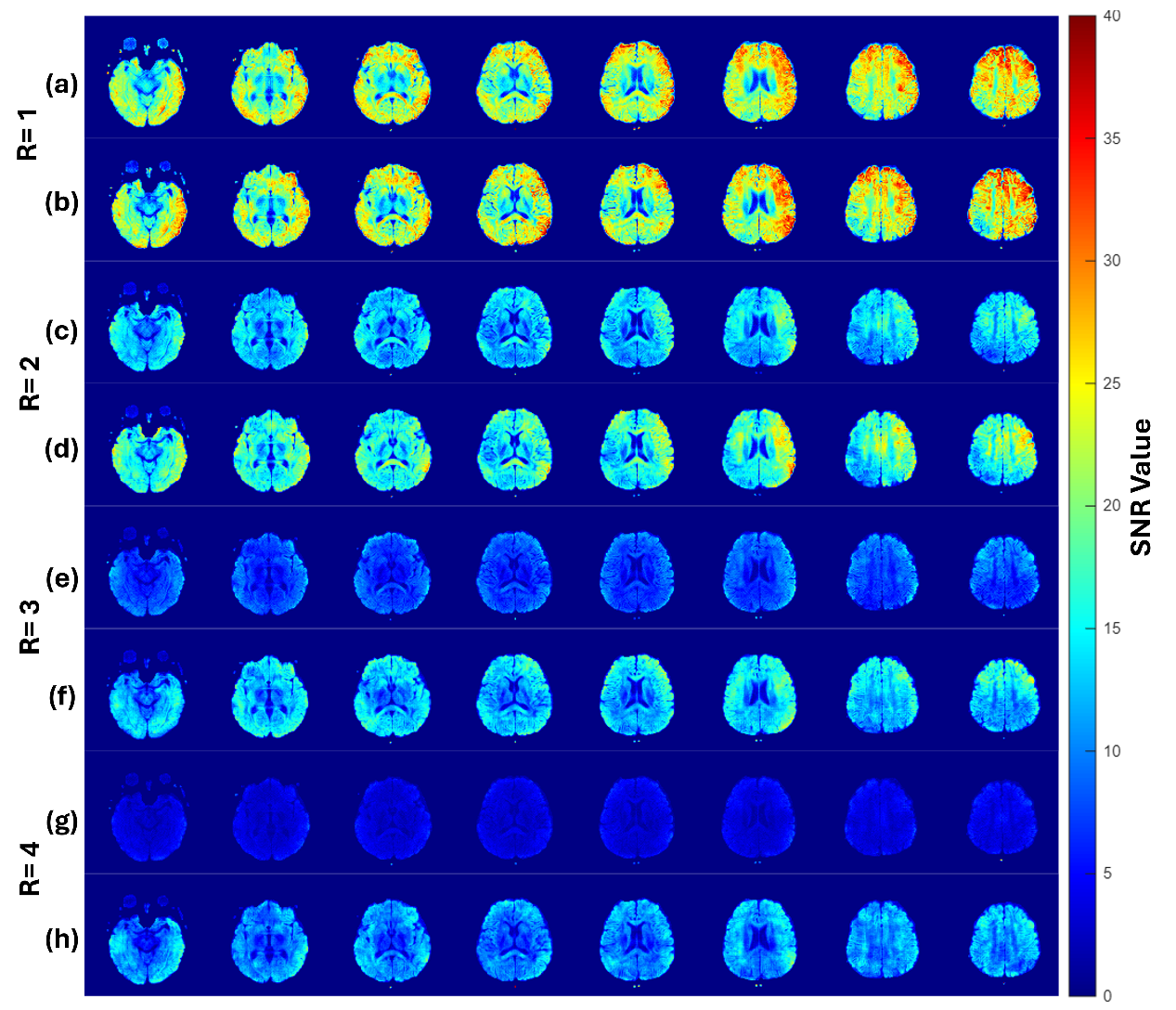


**Figure S6.** The comparison of SNR maps between conventional PROPELLER-EPI reconstruction (a, c, e, g) and the CORPUSE framework (b, d, f, h) for 8-blade LAP-EPI data under different per-blade acceleration factors (R=1 to 4). Columns correspond to eight matched slice locations. CORPUSE consistently yields higher and more spatially uniform SNR across slices, with increasingly pronounced improvements at higher acceleration factors.


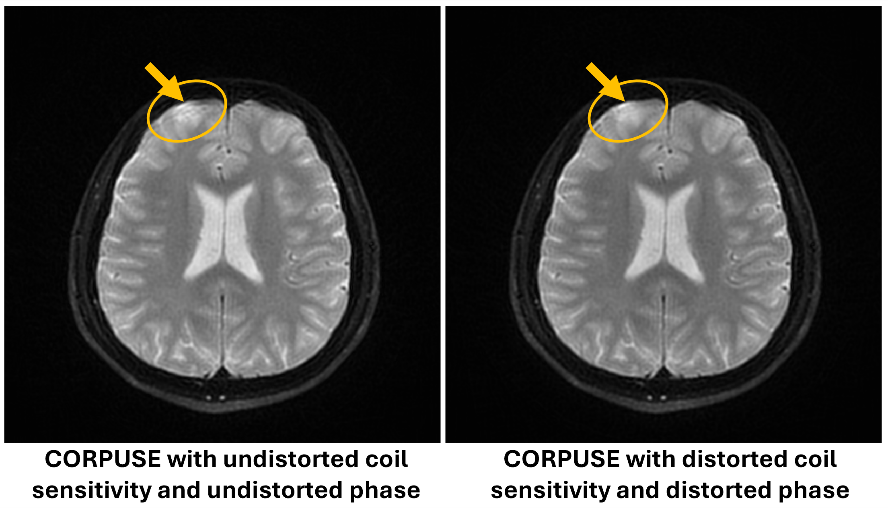


**Figure S7.** Effect of distortion matching in coil sensitivity profiles for CORPUSE reconstruction. Coil sensitivity maps were estimated from undistorted GRE reference data. For comparison, CORPUSE reconstruction was performed using either the original undistorted coil sensitivity maps or blade-specific distortion-matched coil sensitivity maps obtained by warping the reference maps with the corresponding field map. Reconstruction using undistorted coil sensitivity maps and phase information (left) leads to spatial mismatch between the coil sensitivity maps and the distorted PROPELLER-EPI blade, resulting in localized artifacts (arrow). When distortion-matched coil sensitivity maps and corresponding phase information are used (right), geometric consistency between the sensitivity profiles and blade images is preserved, yielding improved reconstruction quality.

**
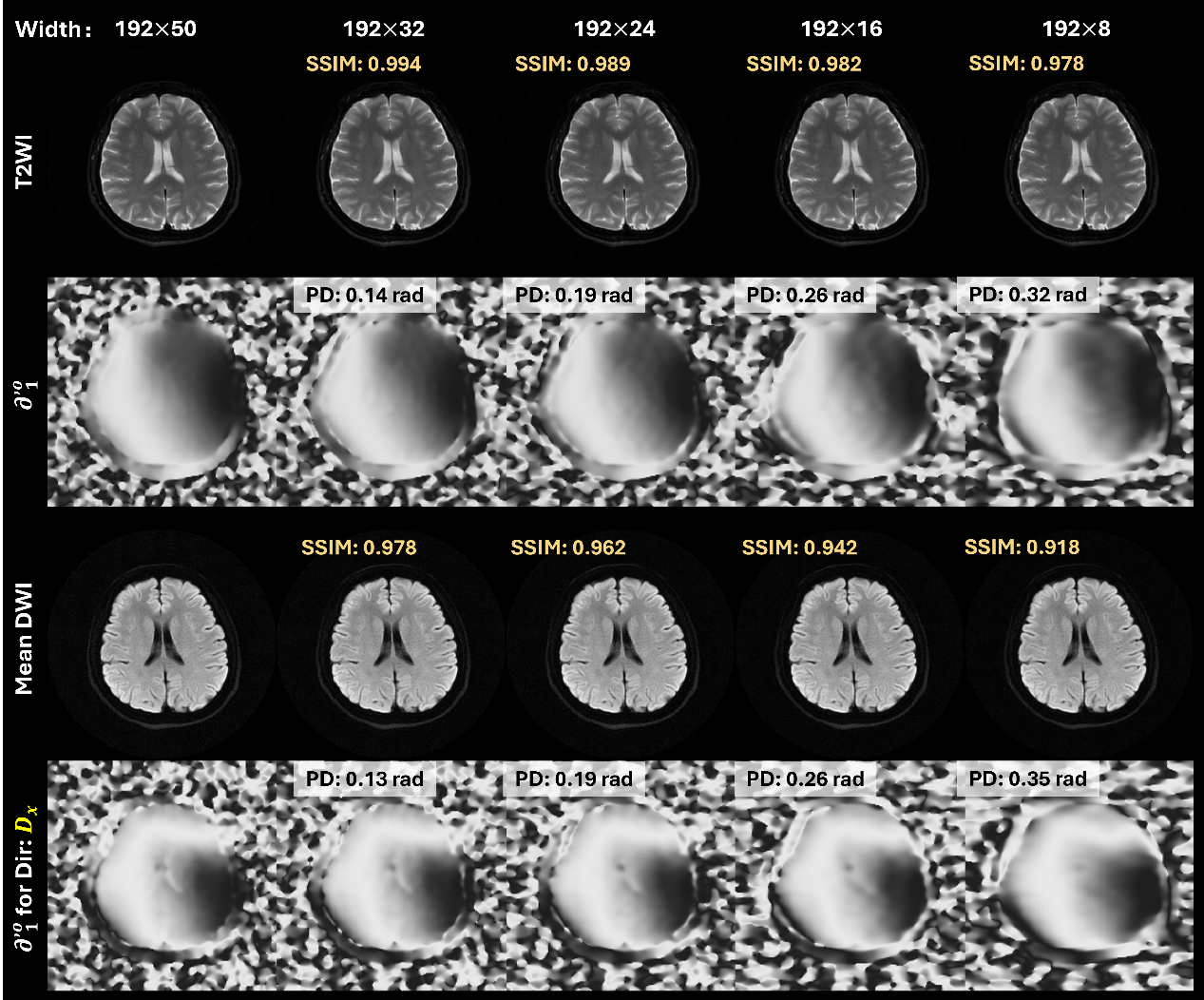
**

**Figure S8.** Parameter experiment evaluating the effect of phase term estimation from different blade widths, while keeping the field map fixed to that estimated from the reference 192×50 blades. Reduced blade widths (192×32, 192×24, 192×16, and 192×8) were retrospectively generated from the original 24-blade dataset, and CORPUSE reconstruction was repeated by replacing only the phase terms estimated from each reduced-width blade. Rows 1 and 3 show the CORPUSE reconstructed T2-weighted and mean DWI images, respectively. Rows 2 and 4 show representative odd echo phase maps for phase correction from the T2 and x-direction diffusion data. PD denotes the mean absolute wrapped phase difference, averaged over all 24 blades, relative to the 192×50 reference reconstruction. SSIM denotes the structural similarity index relative to the 192×50 reference. As blade width decreases, phase-map deviation increases progressively and is accompanied by a monotonic reduction in reconstruction similarity.

**
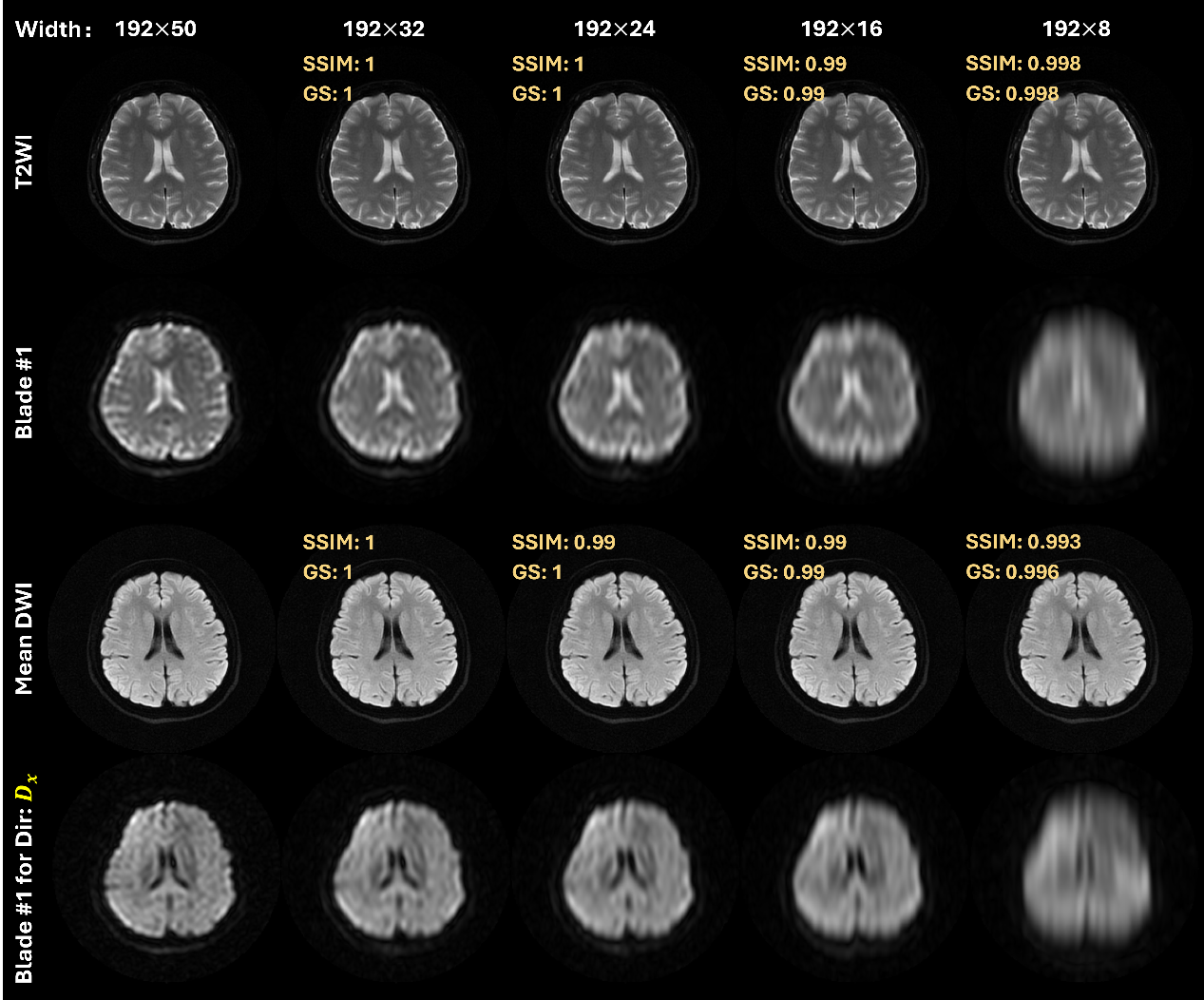
**

**Figure S9.** Parameter experiment evaluating the effect of field map estimation from different blade widths, while keeping the phase map fixed to that estimated from the reference 192×50 blades. Reduced blade widths (192×32, 192×24, 192×16, and 192×8) were retrospectively generated from the original 24-blade dataset, and CORPUSE reconstruction was repeated by replacing only the field maps estimated from each reduced-width blade.Rows 1 and 3 show the CORPUSE reconstructed T2-weighted and mean DWI images, respectively. Rows 2 and 4 show representative reduced-width blades from the T2 and x-direction diffusion data used for field-map estimation. SSIM and GS denote the structural similarity index and gradient similarity, respectively, relative to the 192×50 reference reconstruction.

**
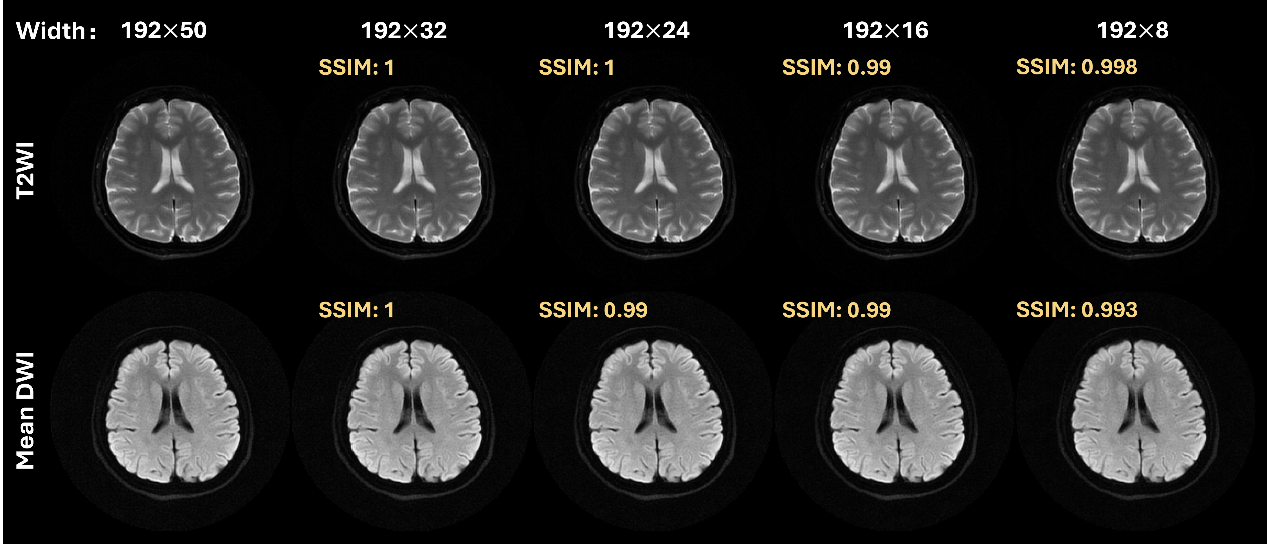
**

**Figure S10.** Parameter experiment evaluating the combined effect of phase map and field map estimation from different blade widths. Reduced blade widths (192×32, 192×24, 192×16, and 192×8) were retrospectively generated from the original 24-blade dataset, and CORPUSE reconstruction was repeated using both the phase maps and field maps estimated from each reduced-width blade. Rows 1 and 2 show the CORPUSE reconstructed T2-weighted and mean DWI images, respectively. SSIM denotes the structural similarity index of the reconstructed images relative to the 192×50 reference reconstruction.
