# Supplementary material for: Collaborative Reconstruction of PROPELLER‐EPI Data Using POCSMUSE (CORPUSE) for High‐Fidelity Diffusion MRI
Source: Magn Reson Med. 2026 May 30;96(4):1929–46. doi: 10.1002/mrm.70448 (PMC13421002; doi:10.1002/mrm.70448)
Supplement: Supplementary file 2 — Table S1: Primary scan parameters for Short‐Axis DW‐PROPELLER‐EPI acquisitions. Table S2: SNR and SNR efficiency comparison of the PROPELLER‐EPI method and proposed CORPUSE method. [file MRM-96-1929-s001.docx]

**Supplementary Table S1.** Primary scan parameters for Short-Axis-DW- PROPELLER-EPI acquisitions.

| **Scan** | **1** | **2** | **3** | **4** | **5** | **6** | **7** |
| --- | --- | --- | --- | --- | --- | --- | --- |
| **# of blades (**$\boldsymbol{N}_{\boldsymbol{b}}$**)** | 12 | 12 | 12 | 12 | 12 | 18 | 6 |
| **Rotation Angle (**$\boldsymbol{\theta}$**)** | 30$^{\circ}$ | 30$^{\circ}$ | 30$^{\circ}$ | 30$^{\circ}$ | 30$^{\circ}$ | 20$^{\circ}$ | 60$^{\circ}$ |
| **FOV (cm)** | 24 | 24 | 24 | 24 | 24 | 24 | 24 |
| **Blade Size (**$\boldsymbol{N\times L}$**)** | 50$\times$192 | 50$\times$192 | 50$\times$192 | 34$\times$192 | 72$\times$192 | 34$\times$192 | 72$\times$192 |
| **PF factor** | 0.63 | 0.63 | 0.63 | 0.63 | 0.63 | 0.63 | 0.63 |
| **ETL** | 120 | 60 | 40 | 40 | 40 | 40 | 40 |
| **Acceleration factor (R)** | 1 | 2 | 3 | 3 | 3 | 3 | 3 |
| **TE (ms)** | 75.4 | 67.7 | 66 | 66 | 66.6 | 66 | 67.7 |
| **b-values (s/mm^2^)** | 0, 1000 | 0, 800 | 0, 1000 | 0, 1000 | 0, 1000 | 0, 1000 | 0, 1000 |
| **# diffusion direction for b-value > 0 s/mm^2^** | 3 | 3 | 3 | 3 | 3 + 1**^*^** | 3 + 1**^*^** | 3 + 1**^*^** |
| **Scan Time^**^ (s)** | 36 | 36 | 36 | 36 | 36 | 54 | 18 |

*Note*: ^*^3 + 1 diffusion direction means first scan with 3 diffusion directions and a repeated scan for 1 diffusion direction.

^**^Scan Time for each diffusion direction.

**Supplementary Table S2**. SNR and SNR efficiency comparison of the PROPELLER-EPI method and proposed CORPUSE method

|  | **Description** | **# of slices** | **Reconstruction Method** | **Quantitative metrics** |
| --- | --- | --- | --- | --- |
| 1 | To compare the SNR and SNR efficiency under different number of blades. | 22 | PROPELLER for 24 Blades LAP-EPI (#Reference)  CORPUSE for 24 Blades LAP-EPI  PROPELLER for 18 Blades LAP-EPI  CORPUSE for 18 Blades LAP-EPI  PROPELLER for 12 Blades LAP-EPI  CORPUSE for 12 Blades LAP-EPI  PROPELLER for 6 Blades LAP-EPI  CORPUSE for 6 Blades LAP-EPI | SNR = 51.52 $\pm$ 4.57, SNR Eff. = 100.00%  SNR = 54.05 $\pm$ 4.07, SNR Eff. = 104.92%  SNR = 43.29 $\pm$ 4.33, SNR Eff. = 97.04%  SNR = 45.30 $\pm$ 4.34, SNR Eff. = 101.55%  SNR = 31.05 $\pm$ 2.73, SNR Eff. = 85.21%  SNR = 35.73 $\pm$ 5.72, SNR Eff. = 98.10%  SNR = 25.03 $\pm$ 2.52, SNR Eff. = 97.17%  SNR = 27.36 $\pm$ 4.89, SNR Eff. = 106.23% |
| 2 | To compare the SNR and SNR efficiency under different acceleration factor for 8-blade data. | 22 | PROPELLER for 24 Blades LAP-EPI (#Reference)  PROPELLER for 8 Blades LAP-EPI (R=1)  CORPUSE for 8 Blades LAP-EPI (R=1)  PROPELLER for 8 Blades LAP-EPI (R=2)  CORPUSE for 8 Blades LAP-EPI (R=2)  PROPELLER for 8 Blades LAP-EPI (R=3)  CORPUSE for 8 Blades LAP-EPI (R=3)  PROPELLER for 8 Blades LAP-EPI (R=4)  CORPUSE for 8 Blades LAP-EPI (R=4) | SNR = 51.52 $\pm$ 4.57, SNR Eff. = 100.00%  SNR = 20.49 $\pm$ 1.84, SNR Eff. = 68.88%  SNR = 20.71 $\pm$ 1.92, SNR Eff. = 69.62%  SNR = 12.57 $\pm$ 1.04, SNR Eff. = 42.24%  SNR* = 16.04 $\pm$ 1.43, SNR Eff. = 53.92%  SNR = 7.24 $\pm$ 0.45, SNR Eff. = 24.36%  SNR* = 12.06 $\pm$ 0.89, SNR Eff. = 40.56%  SNR = 3.19 $\pm$ 0.25, SNR Eff. = 10.73%  SNR* = 9.89 $\pm$ 0.89, SNR Eff. = 33.25% |
| 3 | To evaluate the SNR for SAP-EPI data under different number of blades. | 6 | PROPELLER for 18 Blades SAP-EPI (R=3)  CORPUSE for 18 Blades SAP-EPI (R=3)  PROPELLER for 12 Blades SAP-EPI (R=3)  CORPUSE for 12 Blades SAP-EPI (R=3)  PROPELLER for 6 Blades SAP-EPI (R=3)  CORPUSE for 6 Blades SAP-EPI (R=3) | SNR = 31.16 $\pm$ 2.84  SNR* = 42.50 $\pm$ 3.54  SNR = 21.59 $\pm$ 2.18  SNR* = 30.33 $\pm$ 2.77  SNR = 14.37 $\pm$ 0.94  SNR = 18.64 $\pm$ 2.09 |

*Note*: SNR are shown in (means $\pm$ SD), and SNR Eff. represents SNR Efficiency calculated using 24 Blades data as the reference standard (#Reference).

* denote there is significant difference between different reconstruction methods (p-value<0.001)
